# A global regulatory system links virulence and antibiotic resistance to envelope homeostasis in *Acinetobacter baumannii*

**DOI:** 10.1371/journal.ppat.1007030

**Published:** 2018-05-24

**Authors:** Edward Geisinger, Nadav J. Mortman, Germán Vargas-Cuebas, Albert K. Tai, Ralph R. Isberg

**Affiliations:** 1 Department of Molecular Biology and Microbiology, Tufts University School of Medicine, Boston, Massachusetts, United States of America; 2 Howard Hughes Medical Institute, Boston, Massachusetts, United States of America; 3 Department of Integrative Physiology and Pathobiology, Tufts University School of Medicine, Boston, Massachusetts, United States of America; Case Western Reserve University School of Medicine, UNITED STATES

## Abstract

The nosocomial pathogen *Acinetobacter baumannii* is a significant threat due to its ability to cause infections refractory to a broad range of antibiotic treatments. We show here that a highly conserved sensory-transduction system, BfmRS, mediates the coordinate development of both enhanced virulence and resistance in this microorganism. Hyperactive alleles of BfmRS conferred increased protection from serum complement killing and allowed lethal systemic disease in mice. BfmRS also augmented resistance and tolerance against an expansive set of antibiotics, including dramatic protection from β-lactam toxicity. Through transcriptome profiling, we showed that BfmRS governs these phenotypes through global transcriptional regulation of a post-exponential-phase-like program of gene expression, a key feature of which is modulation of envelope biogenesis and defense pathways. BfmRS activity defended against cell-wall lesions through both β-lactamase-dependent and -independent mechanisms, with the latter being connected to control of lytic transglycosylase production and proper coordination of morphogenesis and division. In addition, hypersensitivity of *bfmRS* knockouts could be suppressed by unlinked mutations restoring a short, rod cell morphology, indicating that regulation of drug resistance, pathogenicity, and envelope morphogenesis are intimately linked by this central regulatory system in *A*. *baumannii*. This work demonstrates that BfmRS controls a global regulatory network coupling cellular physiology to the ability to cause invasive, drug-resistant infections.

## Author summary

Infections with the hospital-acquired bacterium *Acinetobacter baumannii* are highly difficult to treat. The pathogen has evolved multiple lines of defense against antimicrobial stress, including a barrier-forming cell envelope as well as control systems that respond to antimicrobial stresses by enhancing antibiotic resistance and virulence. Here, we uncovered the role of a key stress-response system, BfmRS, in controlling the transition of *A*. *baumannii* to a state of heightened resistance and virulence. We show that BfmRS enhances pathogenicity in mammalian hosts, and augments the ability to grow in the presence of diverse antibiotics and tolerate transient, high-level antibiotic exposures. Connected to these effects is the ability of BfmRS to globally reprogram gene expression and control multiple pathways that build, protect, and shape the cell envelope. Moreover, we determined that resistance-enhancing mutations bypassing the need for BfmRS also modulate envelope- and morphology-associated pathways, further linking control of physiology with resistance in *A*. *baumannii*. This work uncovers a global control circuit that shifts cellular physiology in ways that promote hospital-associated disease, and points to inhibition of this circuit as a potential strategy for disarming the pathogen.

## Introduction

Infections with multidrug-resistant (MDR) bacteria pose a serious threat to public health. The ability to withstand multiple antibiotic treatments derives from a diverse array of genetic resistance determinants, many of which are deeply intertwined with aspects of microbial physiology including virulence [1, 2]. Understanding how intrinsic resistance systems are controlled and how they modulate pathogen-host interactions promises to reveal ways to target MDR bacteria.

An MDR pathogen that has emerged as a major cause of recalcitrant opportunistic infections in hospitals worldwide is *Acinetobacter baumannii*. *A*. *baumannii* is a Gram-negative rod that has evolved high rates of resistance to a broad array of antibiotics. Nosocomial diseases caused by *A*. *baumannii* are notoriously difficult to treat and manifest commonly as bacteremia, pneumonia, wound infections, and sepsis [3].

Modeling pathogenesis with *A*. *baumannii* has presented challenges. Immunocompetent mouse model hosts typically show resistance to the development of lethal disease upon infection with *A*. *baumannii* [3]. A number of bacterial products, many of which involve the multilayered cell envelope [4], have been shown to promote fitness in hosts and also contribute to intrinsic drug resistance [3, 5–7]. How the production of these factors is controlled by the pathogen remains poorly characterized. Understanding this control circuitry is complicated by the fact that *Acinetobacter* lacks several canonical systems that regulate antimicrobial stress responses in other organisms [8].

Our previous work has uncovered the existence of a regulatory network that responds to antibiotic stress and controls virulence in *A*. *baumannii* [5]. We found that production of an important protective component of the *A*. *baumannii* envelope, capsular exopolysaccharide, is transcriptionally upregulated by the antibiotic chloramphenicol at sub-minimal inhibitory concentration (MIC) [5]. Such treatment causes a dramatic phenotypic conversion of *A*. *baumannii* from an avirulent state to one that can cause lethal disease in mice [5]. Signaling from a highly conserved two-component system (TCS), BfmRS, is required for transcriptional upregulation of capsule in response to antibiotics [5]. The phenotypes of *bfmS* and *bfmR* null mutants fit a model in which the BfmS receptor histidine kinase negatively regulates its cognate response regulator BfmR by altering its phosphorylation state [5].

BfmRS is critical for various functions relevant to nosocomial disease. Bacteria lacking BfmR show reductions in biofilm production [9], resistance to certain antibiotics [10], survival in human ascites fluid and serum [10], and fitness in animal hosts [7, 11]. In a mouse *A*. *baumannii* pneumonia model, *bfmR* mutants are the most severely attenuated within a saturating genomic transposon mutant pool, consistent with the BfmR-controlled regulatory network playing a central role in the disease process [11]. Moreover, *bfmR* mutants exhibit elongated morphologies [5, 9], and *bfmS* knockout allows bypass of toxicity due to a late-stage capsule biosynthesis defect [5], consistent with roles for this system in envelope biogenesis. Despite its importance, we understand little about how BfmRS regulates gene expression, and how such control influences antibiotic resistance and the ability of the pathogen to cause opportunistic disease.

Here, we have addressed these questions by analyzing the role of BfmRS in jointly promoting broad-range drug resistance, defense from host attack, and bacterial physiology. This analysis reveals that transcriptional control of cell envelope morphogenesis is fundamentally connected to both intrinsic antibiotic resistance and pathogenic potential in *A*. *baumannii*.

## Results

### Activation of the BfmRS Two component system enhances virulence

**Fig 1:**
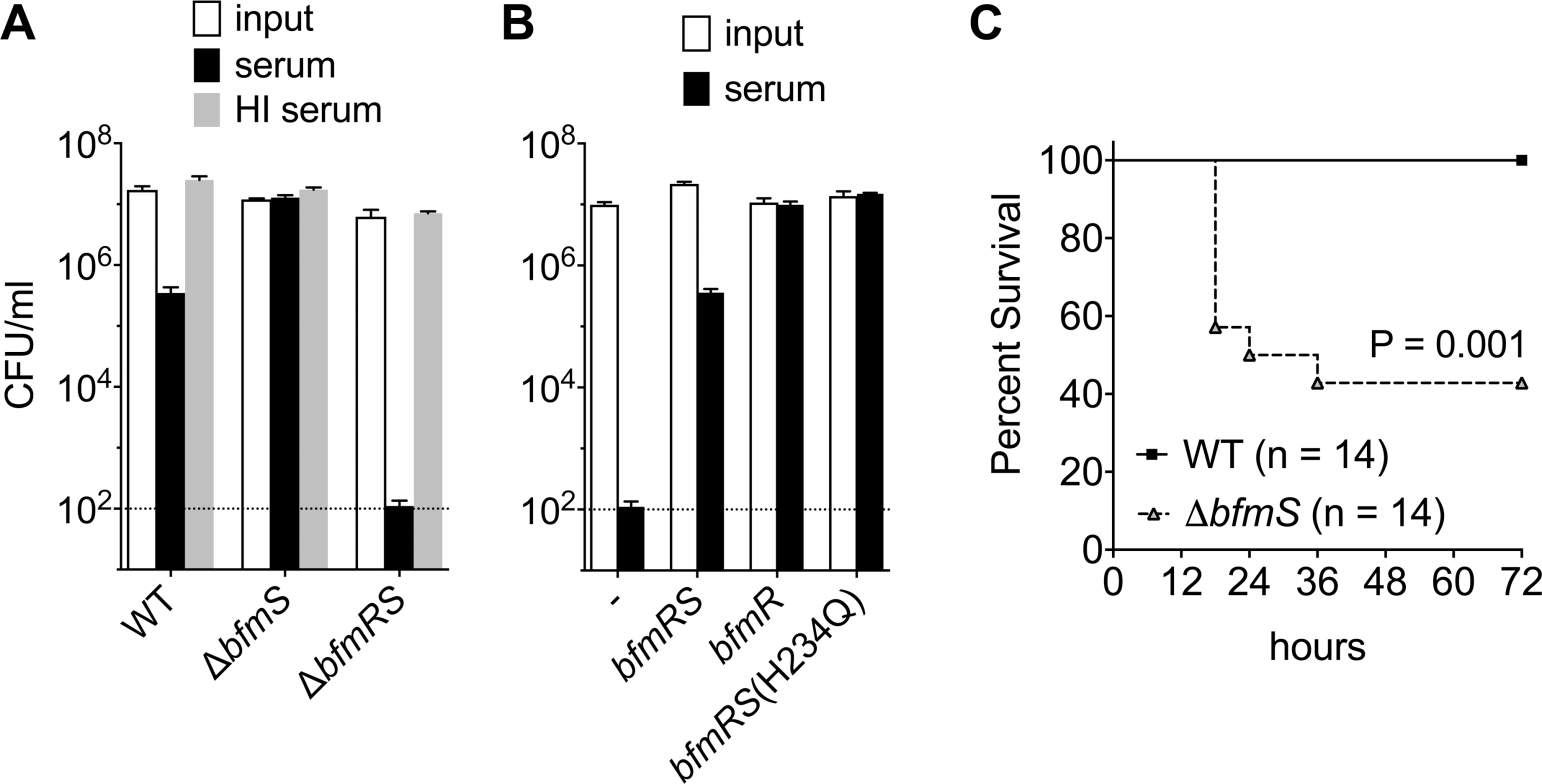
Activation of the BfmRS TCS enhances virulence-associated functions. (A-B) Modulation of serum resistance by BfmRS. Strains were incubated in baby rabbit serum or heat-inactivated serum for 1 hr. The number of viable bacteria before (input) and after incubation in serum was determined by quantitating CFU on LB agar (Materials and Methods). Lines show mean ± s.d. (n = 4). The limit of detection is indicated by the dotted line. Strains tested were 17978 (WT), EGA195 (Δ*bfmS*), or EGA495 (Δ*bfmRS*) (A); and EGA495 (Δ*bfmRS*) carrying no additional construct (-) or the indicated *bfmRS* allele in single copy (B). (C) Δ*bfmS* bacteria show enhanced virulence in a mouse model of systemic infection. Mice were challenged via intraperitoneal delivery of approximately 10^8^ bacteria, and survival was monitored. Mean CFU in the inocula were 1.01x10^8^ (WT) and 7.5x10^7^ (Δ*bfmS*). Survival data are pooled from two independent experiments. Statistical significance was determined by the Gehan-Breslow-Wilcoxon Test.

We previously found that antibiotics that stimulate capsule hyper-production augment the ability of *A*. *baumannii* to cause lethal systemic disease [5]. As BfmRS activity is necessary and sufficient for capsule hyper-production [5], we hypothesized that activation of BfmRS signaling would similarly result in augmented virulence. To test this model, we measured serum killing and virulence of strains carrying *bfmRS* mutant alleles that are predicted to activate or abolish the TCS control circuit. Exposure of 60% rabbit serum for 1 hour reduced viable counts of the wild-type (WT) strain approximately 50-fold (Fig 1A). Deletion of *bfmS*, predicted to activate the control circuit, resulted in complete resistance to killing by serum (Fig 1A), phenocopying the high-level serum resistance produced by antibiotic pretreatment [5]. In contrast, loss of the TCS due to deletion of *bfmRS* resulted in efficient killing, consistent with a prior study [10], with survival levels approximately 10^−5^ relative to the *bfmS* deletion (Fig 1A). Killing was dependent on complement activity because no reductions in viability were observed with heat-inactivated serum (Fig 1A). The altered complement susceptibility phenotypes of *bfmRS* mutants were specific to this locus; reintroduction of WT *bfmRS* into a Δ*bfmRS* strain produced levels of serum susceptibility that were similar to WT, while reintroduction of *bfmR* in the absence of *bfmS* or in the presence of a catalytically inactive *bfmS* allele [*bfmS*(H234Q)] recapitulated the high-level resistance to serum seen in the hyperactive Δ*bfmS* strain (Fig 1B).

Enhanced serum complement resistance is tightly connected to increased virulence potential of *A*. *baumannii* in mammalian infection models [12–16]. To determine if loss of BfmS function similarly enhances virulence relative to WT, the mutant was tested in a systemic infection model employing immunocompetent mice which normally survive WT infection and restrict the organism [5, 13]. Intraperitoneal infection with the Δ*bfmS* strain caused lethal, septic disease in approximately 60% of the inoculated mice, at a dose that was completely nonlethal with WT bacteria (Fig 1C). These results show that BfmRS controls the ability to develop virulence in *A*. *baumannii* in a manner that mirrors the effects of sub-MIC antibiotic treatment. Interestingly, by contrast with sub-MIC chloramphenicol treatment, which partially inhibits bacterial growth [5], *bfmS* mutation had no discernable effect on culture growth rates or yield, at least by using simple short term broth growth (S1 Fig). The Δ*bfmRS* mutant showed logarithmic phase growth that was similar to WT but produced lower post-exponential phase growth yield (S1 Fig).

### BfmRS confers broad-range antibiotic resistance

**Fig 2:**
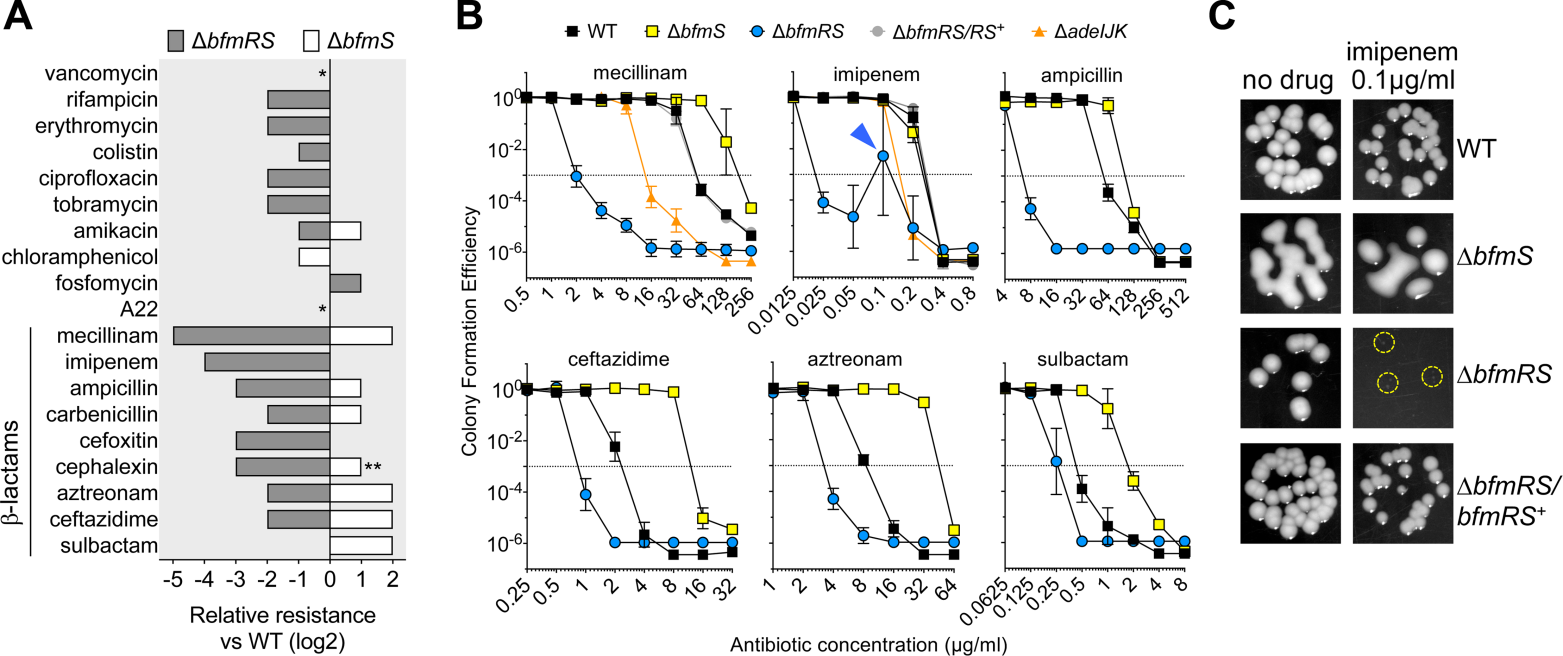
BfmRS confers broad-range antibiotic resistance including pronounced defense against β-lactams. (A) Relative resistance of EGA195 (Δ*bfmS*) or EGA495 (Δ*bfmRS*) compared to WT control. Bacteria were grown on solid medium containing increasing concentrations of antibiotics, and mean CFE was quantified from multiple cultures (n ≥ 2). The lowest concentration at which mean CFE <10^−3^ defines the MIC of each antibiotic, and resistance level relative to WT was calculated as log_2_(MIC_mutant_/MIC_WT_). *, MIC for both mutants was identical to WT. **, With cephalexin, the MIC against Δ*bfmS* was >400μg/ml; the next 2-fold-increased concentration (800μg/ml) was used in calculating relative resistance. (B) CFE with bacteria grown on increasing concentrations of the indicated antibiotics on solid medium. Data points represent the geometric mean ± s.d. (n ≥ 2). Dotted line at CFE = 10^−3^ indicates threshold for MIC determination. Arrowhead indicates presence of pinpoint-sized colonies. In this panel, EGA465 (Δ*adeIJK*) and EGA497 (Δ*bfmRS*/*bfmRS*^+^) were utilized only in tests with mecillinam and imipenem. (C) Images of colonies grown on solid medium from panel *B* imipenem assay. Δ*bfmRS* formed pinpoint-sized colonies (circled) when grown with imipenem 0.1 μg/ml.

Given the ability of BfmRS to modulate bacterial defense against host antimicrobial killing and its described effects on resistance to some antibiotics [6, 10], we determined the ability of this system to control the outcome of a wide range of antibiotic interactions. We first compared *bfmRS* null and hyperactive mutants for their resistance to a set of 19 antibiotics by profiling populations of each strain for their colony formation efficiency (CFE) [5] on medium containing increasing concentrations of antibiotics. From the resulting CFE profiles (shown in Fig 2B and S2A Fig), the MIC of each antibiotic against the different strains was determined (see Materials and Methods and S3 Table). The effects of *bfmRS* mutation on each drug’s MIC are summarized in Fig 2A. We observed that knockout of *bfmRS* caused hypersensitivity to the vast majority of antibiotics tested, and the constitutively active Δ*bfmS* strain caused hyperresistance to a substantial subset (Fig 2A).

Although multiple diverse classes of antibiotics were affected, resistance to the β-lactam class of antibiotics, which targets assembly of the peptidoglycan component of the cell envelope, demonstrated the most pronounced dependency on *bfmRS* activity. Furthermore, in the absence of BfmS, resistance to β-lactams was enhanced for most of the drugs (Fig 2A). In the case of mecillinam and imipenem, MICs of the Δ*bfmRS* strain were 32- and 16-fold lower, respectively, compared to WT (Fig 2A and 2B). As a point of reference, loss of the key AdeIJK efflux system, a major intrinsic resistance determinant, caused a much lower degree of hypersensitivity (4- and 2-fold reductions in MIC with mecillinam and imipenem, respectively, compared to WT; Fig 2B), highlighting the profound defect caused by loss of BfmRS. Surprisingly, barely visible pinpoint colonies of the Δ*bfmRS* strain appeared at high frequency at 0.1 μg/ml of imipenem (Fig 2B, arrowhead, and Fig 2C, circles), even though these were not apparent at lower or higher concentrations. One possible explanation is that a BfmRS-independent stress response triggered by imipenem, but normally masked in the WT strain, became apparent at this drug concentration in the *bfmRS*-null mutant. In the presence of the β-lactams ceftazidime, aztreonam, or sulbactam, the Δ*bfmRS* strain had modestly decreased CFE vs WT while the constitutively active Δ*bfmS* strain had much greater growth (Fig 2A and 2B, S2A Fig). Hypersensitivity and hyperresistance with Δ*bfmRS* and Δ*bfmS*, respectively, were also seen with additional β-lactam antibiotics including ampicillin, carbenicillin, and cephalexin (Fig 2A and 2B, S2A Fig). These resistance phenotypes were not apparent, however, with some envelope-targeting antibiotics, such as the non-β-lactam antibiotic phosphomycin, which inhibits peptidoglycan precursor synthesis [17], or the MreB antagonist A22, which inhibits cell wall synthesis during bacterial elongation [18] (Fig 2A and S2A Fig). This indicates that BfmRS may be particularly protective against the bactericidal properties of β-lactams, which cause cell-wall assembly enzymes to malfunction [19].

The dependence of the observed resistance phenotypes on the function of BfmRS was assessed by recombining a variety of alleles into the Δ*bfmRS* strain at the parental chromosomal site and assaying for CFE with a subset of antibiotics. We observed that reintroduction of WT *bfmRS* rescued the WT phenotype (Fig 2B, S2 Fig, S3 Table). Furthermore, introduction of *bfmR* alone into the Δ*bfmRS* strain increased CFE to levels identical to the Δ*bfmS* strain (S2B Fig). Replacement of Δ*bfmRS* by *bfmR* together with the *bfmS* kinase-defective allele (*bfmS*(H234Q)) also resulted in hyper-resistance (S2B Fig), consistent with the model that BfmS negatively regulates the resistance-promoting activities of BfmR.

### BfmRS controls antibiotic tolerance

**Fig 3:**
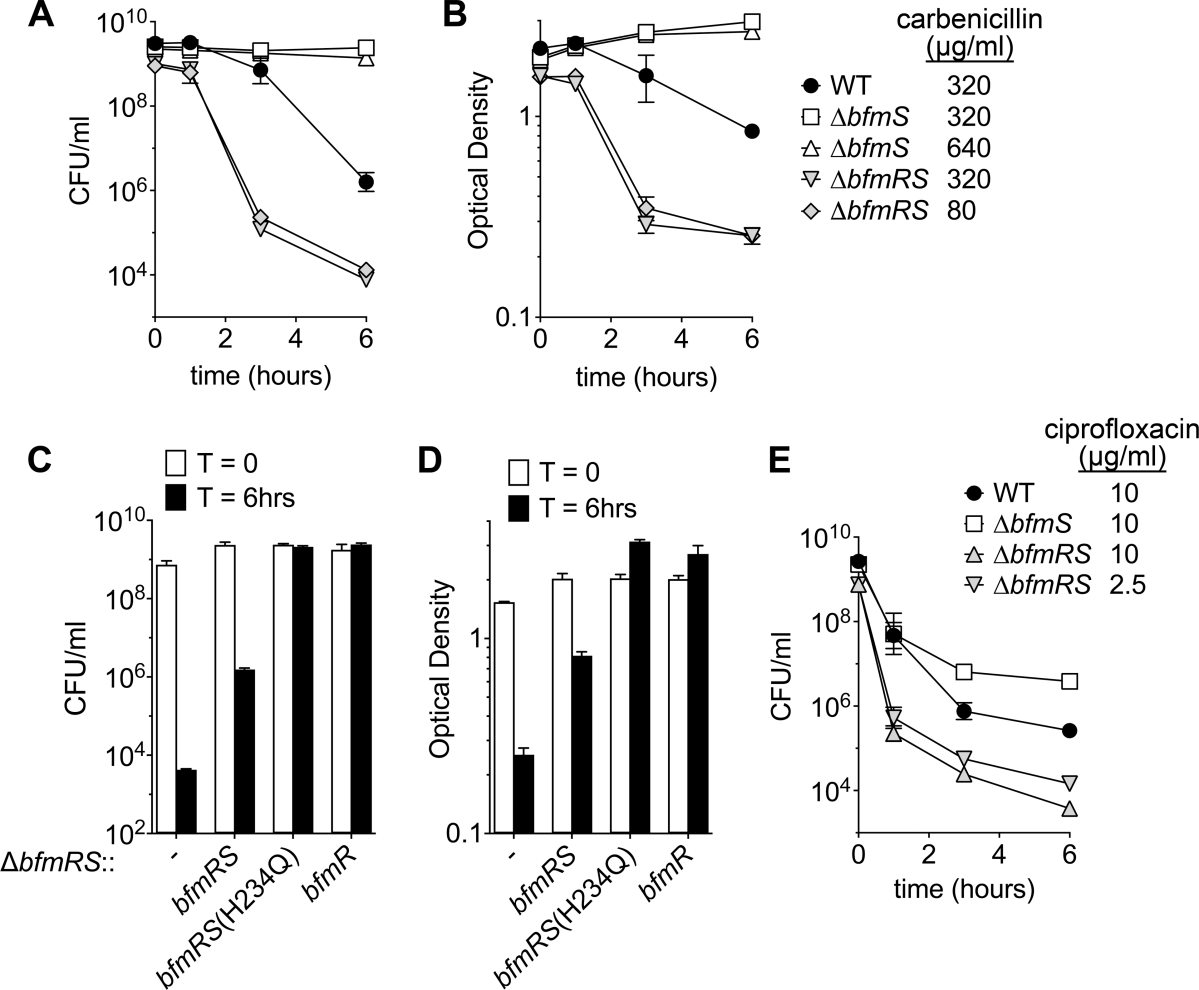
BfmRS controls antibiotic tolerance and persistence. 17978 (WT), EGA195 (Δ*bfmS*), or EGA495 (Δ*bfmRS*) bacteria grown to early-post exponential phase were challenged with the indicated antibiotic at the listed supra-MIC levels. Bacteria sampled at the indicated time points were washed, serially diluted, and plated on solid medium lacking antibiotics to determine viable bacterial counts at each time point (A, C, and E). Culture A_600_ was also determined (B and D). In C and D, EGA495 (Δ*bfmRS*) carrying no additional construct (-) or the indicated *bfmRS* allele in single copy were challenged with carbenicillin at 320 μg/ml. Data points represent mean ± s.d. (n ≥ 2).

Given the enhanced antibiotic resistance and protection from serum killing that resulted from BfmRS activity, we hypothesized that this system would also modulate antibiotic tolerance during transient exposures to high levels of drug. To examine antibiotic tolerance, we measured bacterial viability after challenge with carbenicillin or the fluoroquinolone ciprofloxacin at concentrations well above the MIC (Materials and Methods). With carbenicillin at 320 μg/ml (20X the WT MIC), viability of the WT strain slowly declined over time (Fig 3A). Loss of culture optical density (OD) followed similar kinetics (Fig 3B), consistent with lysis of the killed population. Strikingly, the Δ*bfmS* mutant showed no loss in viability or culture OD at this same concentration or at a higher concentration (640 μg/ml, 20X the MIC of Δ*bfmS*), used to account for the higher MIC compared to WT (Fig 3A and 3B). By contrast, the Δ*bfmRS* mutant showed rapid killing at both 80 μg/ml (20X MIC) or 320 μg/ml equivalently (Fig 3A and 3B). Reintroduction of *bfmRS* or *bfmR* into the Δ*bfmRS* mutant rescued tolerance to the levels of the WT or hyper-resistant strains respectively (Fig 3C and 3D), confirming that these phenotypes were dependent specifically on BfmRS. High-level survival was also observed with expression of *bfmR*^*+*^*bfmS*(H234Q) in the Δ*bfmRS* background (Fig 3C and 3D), consistent with loss of BfmS activity causing increased tolerance. As a consequence of BfmRS function, there was a population-wide decrease in killing by carbenicillin accompanied by an increased duration of drug treatment necessary to drop viability. These results are consistent with of BfmRS function supporting increased antibiotic tolerance [20].

In killing assays employing high concentrations of ciprofloxacin (10μg/ml, or 20X MIC), WT bacteria showed a biphasic time-kill curve, with an initial rapid killing phase followed by a plateau phase between 3 and 6h (Fig 3E) consistent with a sub-population of tolerant cells, or persisters [20]. With this same concentration of drug (which was also 20X MIC of Δ*bfmS*), Δ*bfmS* bacteria showed a rate of killing during the first hour of exposure that was similar to WT followed by a plateau resulting in a persistent population that had at least 10X CFU higher than WT (Fig 3E). After repassaging several surviving Δ*bfmS* clones on medium lacking antibiotics, 100% (16/16) showed levels of ciprofloxacin sensitivity that were unchanged from non-treated parental bacteria, indicating that the enhanced survival rates were not due to mutation. In contrast to Δ*bfmS*, the Δ*bfmRS* mutant had an initial rate of killing that was more rapid than WT and had fewer survivors in the persister phase after treatment with either 10μg/ml or 2.5μg/ml ciprofloxacin, concentrations that are 80X and 20X the MIC of Δ*bfmRS* respectively (Fig 3E). These results demonstrate that BfmRS, in addition to modulating virulence and drug resistance, mediates enhanced drug tolerance in *A*. *baumannii*.

### Global gene expression alterations dependent on BfmRS activity

**Fig 4:**
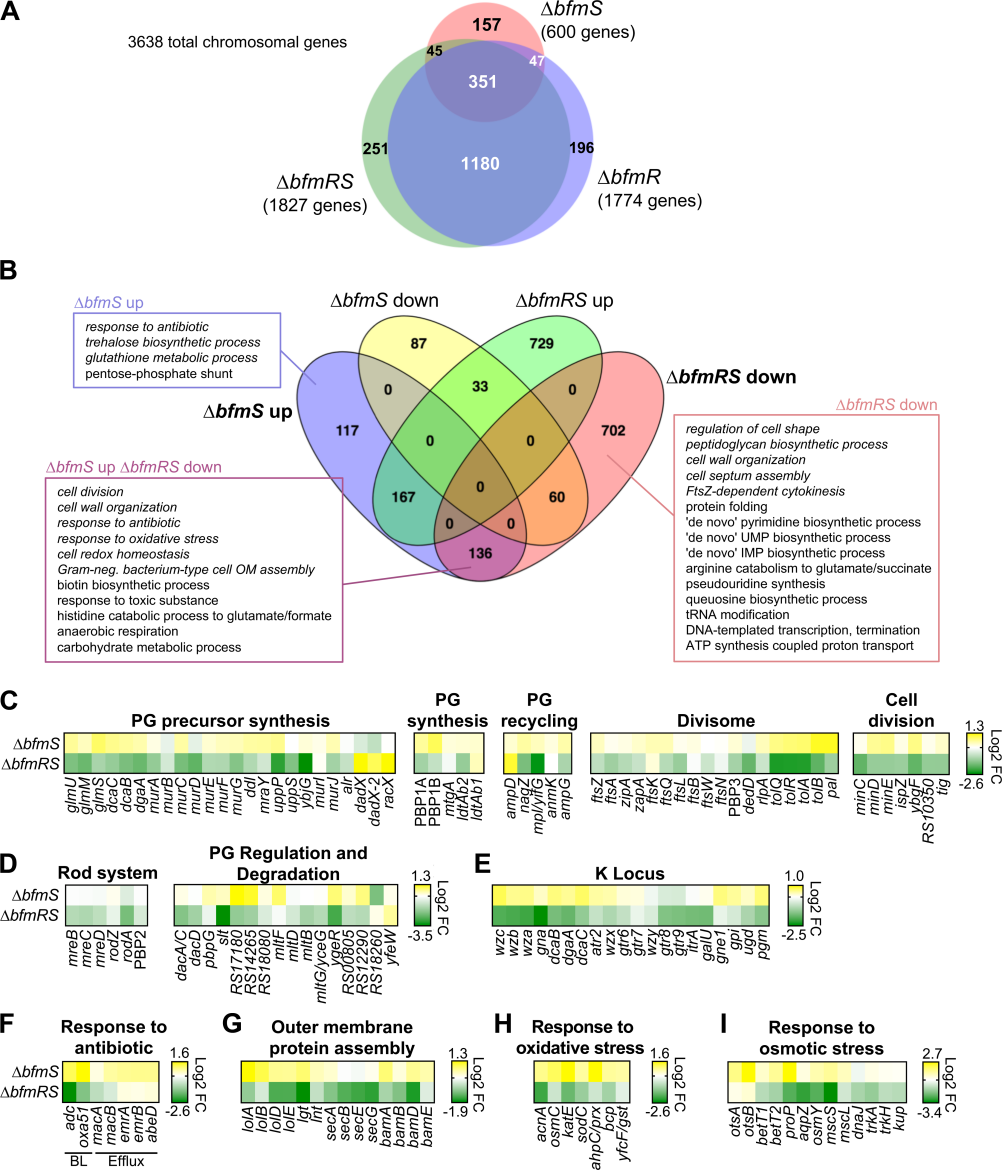
BfmRS globally reprograms the *A*. *baumannii* transcriptome. (A) Venn diagram showing differentially expressed chromosomal genes in each mutant [EGA195 (Δ*bfmS*), EGA496 (Δ*bfmR*), or EGA495 (Δ*bfmRS*)] vs WT. The *A*. *baumannii* chromosome contains 3638 total genes. Differential expression was determined by pairwise comparison of RNAseq data between each mutant and WT, requiring a q value < 0.05 to define a significant change. (B) Gene ontology (GO)-term enrichment analysis of genes differentially expressed due to *bfmRS* mutations. The Venn diagram depicts differentially expressed chromosomal genes whose up- or down-regulation is unique or common to different *bfmRS* alleles. GO biological process terms that were enriched in each category are indicated. Enriched terms required at least 2 genes in the differentially expressed gene subset, >2-fold increase in frequency in the differentially expressed gene set vs the entire genome, and a q value < 0.05. Enriched terms from only Δ*bfmS*-up, Δ*bfmRS*-down, and Δ*bfmS*-up Δ*bfmRS*-down gene subsets are shown. OM, outer membrane. (C-I) Heat maps showing *bfmRS*-dependent differential expression of genes in functional categories related to envelope synthesis and stress. Heat maps show log_2_ fold-change (FC) in transcription of each gene as determined by pairwise comparison of the indicated mutant vs WT via RNAseq. Grouping of genes in each pathway was determined by their associated GO terms and based on orthology with well-characterized *E*. *coli* proteins [21, 62, 63]. PG, peptidoglycan; BL, β-lactamases.

To identify transcriptional activity dependent on BfmRS function, RNA-seq analysis was performed on *bfmRS* mutants and the isogenic WT strain. Comparing Δ*bfmRS* with WT, 1827 chromosomal genes (out of 3638) were differentially expressed based on a false-discovery rate (q value) < 0.05 (Fig 4A and S1 Dataset). Deletion of *bfmR* alone resulted in 1774 genes showing altered expression, the majority of which overlapped with those altered in Δ*bfmRS* (1531 genes overlapping out of a total set of 2070, or 74%) (Fig 4A and S2 Dataset). This is consistent with the model that BfmS signaling is dependent on its cognate response regulator BfmR, and the fact that the two *bfmR* mutants are phenotypically similar (S3 Table) [5]. Comparing Δ*bfmS* with WT, 600 chromosomal genes were differentially expressed (Fig 4A and S3 Dataset), and these mostly (~74%; 443 genes) overlapped with those whose expression was modified in the Δ*bfmRS* and Δ*bfmR* strains. Our *A*. *baumannii* reference strain (ATCC 17978) contains 3 plasmids, and transcript levels of many plasmid genes were also affected (S3A Fig), with Δ*bfmS* by-and-large decreasing expression of plasmid genes relative to WT (S3B Fig). Together these results demonstrate that largescale changes in gene expression occur with change in BfmRS alleles.

To link transcriptional reprogramming by BfmRS to function, we performed pathway enrichment analysis (Materials and Methods), identifying the enriched Gene Ontology (GO) terms (Fig 4B). Given the opposing phenotypes of Δ*bfmS* (UP) and Δ*bfmRS* (DOWN) mutants, we focused our analysis on the gene subsets whose transcription was up in Δ*bfmS* or down in Δ*bfmRS* (Fig 4B, subsets indicated by boxes). In these gene subsets we observed enrichment of several processes related to the cell envelope (Fig 4B, italicized terms). Based on the enrichment of cell wall growth, division, and morphogenesis terms, we examined the full set of proteins in *A*. *baumannii* involved in these pathways that had orthologs in *E*. *coli* [21]. We found that many of the genes in these pathways were under the coordinated control of BfmRS (Fig 4C and 4D), as were the two chromosomally-encoded β-lactamases (the AmpC-type cephalosporinase ADC, and the class D β-lactamase OXA-51) linked to the “response to antibiotic” term (UP in Δ*bfmS* and DOWN in Δ*bfmRS*; Fig 4F). Our analysis found that outer membrane protein assembly pathways were also influenced by the state of BfmRS activity (Fig 4G). Consistent with previous work [5], expression of K locus genes responsible for capsule and other envelope glycans [14] was also altered as a result of *bfmRS* mutation (Fig 4E). These findings indicate that global transcriptome modulation by BfmRS involves control of processes central to envelope homeostasis, consistent with a role in resistance to envelope-targeting antibiotics.

Further analysis indicated that BfmRS modulates *A*. *baumannii* stress responses, particularly at the level of the cell envelope. These include responses to oxidative and osmotic stresses (Fig 4H and 4I), as well as several drug efflux transporters that were modestly up-regulated as a result of the Δ*bfmS* allele (Fig 4F). Multiple metabolic pathways also had altered regulation as a result of *bfmRS* mutation; this was the case in gene subsets with lowered transcription in Δ*bfmRS*, increased in Δ*bfmS*, or both (Fig 4B) as well as in other gene subsets (S4 Fig).

**Fig 5:**
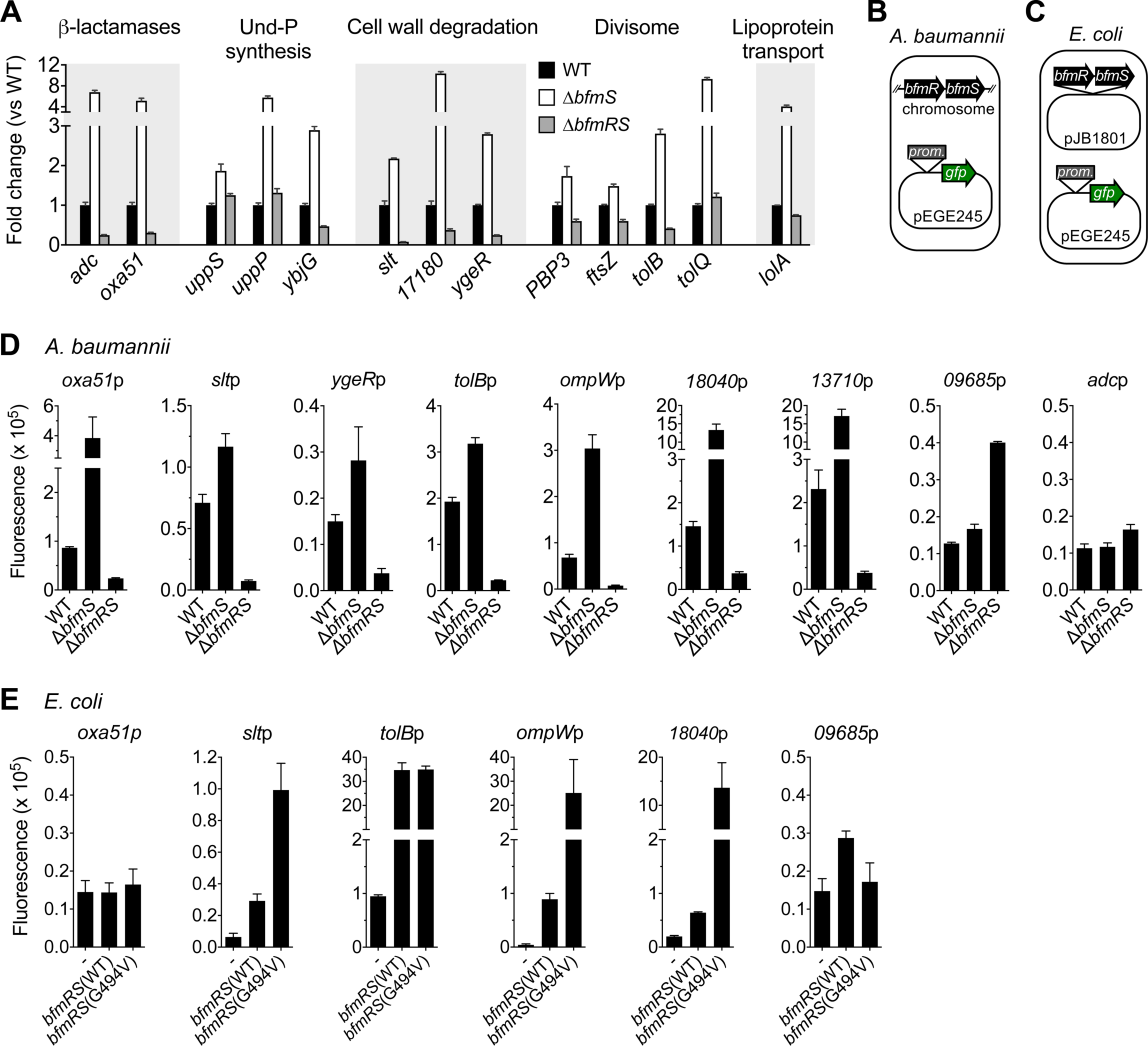
Transcriptional regulation by BfmRS assessed by qRT-PCR and reporter analysis. (A) RNA from the indicated strains was reverse-transcribed and probed via qPCR with primers specific for genes encoding proteins linked to the indicated functional category. Shown is the mean of the fold change in transcript levels (vs WT) ± s.d. (n = 3). For each gene, P < 0.02 in unpaired t-tests comparing mutant vs WT. 17180 denotes ACX60_RS17180, a predicted lytic transglycosylase. (B-E) Analysis of BfmRS control of gene expression by using transcriptional reporter fusions. Strains were grown to early post-exponential phase, and reporter activity measured in microtiter format (Materials and methods). (D) *A*. *baumannii* strains 17978 (WT), EGA195 (Δ*bfmS*), or EGA495 (Δ*bfmRS*) contained a derivative of plasmid pEGE245 encoding GFP transcriptional fusions to the promoter regions upstream of the indicated gene, as diagramed in panel *B*. 18040, 13710, and 09685 denote ACX60_RS18040, ACX60_RS13710, and ACX60_RS09685, respectively. (E) *E*. *coli* DH5α harbored pEGE245 reporter derivatives as well as compatible plasmid pJB1801 containing no additional gene (-) or the indicated *bfmRS* allele, as diagramed in panel *C*. Reporter fluorescence was calculated as the fluorescence units/A_600_ of the reporter bacteria minus the fluorescence units/A_600_ of control bacteria containing vector alone. Bars show mean ± s.d. (n = 3). In unpaired t-tests comparing mutant vs WT (D) or *bfmRS* allele vs vector alone (E), P < 0.04 for each comparison except for: *adc*p [Δ*bfmS* vs WT], *oxa51*p [*bfmRS* vs vector and *bfmRS*(G494V) vs vector], and 09685p [*bfmRS*(G494V) vs vector] (P >0.5).

To validate our RNA-seq analysis, we measured the effect of BfmRS on transcription of genes associated with various envelope processes by using qPCR and transcriptional reporter fusions. We focused our qPCR analysis on genes encoding β-lactamases, synthases of the lipid carrier undecaprenyl-phosphate (Und-P), proteins involved in cell wall degradation and cell septation, and a key lipoprotein biogenesis protein. Transcription levels of each of these genes were consistent with the RNAseq results, with significant up-regulation in the Δ*bfmS* strain in all cases, and down-regulation in the Δ*bfmRS* strain with most of the genes (Fig 5A). To determine whether BfmRS regulates transcription initiation, we constructed transcriptional fusions of a GFP reporter (Fig 5B) to the promoter regions of several genes predicted by transcriptome analysis to be BfmRS-regulated and whose products function in the cell envelope. Reporter fusions to promoter regions upstream of *oxa51*, *slt* (periplasmic lytic transglycosylase responsible for cell wall turnover [18]), *ygeR* (LytM domain, predicted to control daughter cell separation [22]), *tolB*, *ompW* (outer membrane protein), ACX60_RS18040 (predicted lipoprotein), and ACX60_RS13710 (membrane glycoprotein [23]) each demonstrated higher activity in Δ*bfmS* and lower activity in Δ*bfmRS A*. *baumannii* cells compared to WT (Fig 5D). By contrast, GFP fusion to the promoter of a gene not displaying BfmRS-dependent enhancement of transcription in our RNA-seq analysis (ACX60_RS09685, predicted iron acquisition protein) resulted in highest activity in the Δ*bfmRS* mutant (Fig 5D). GFP fusion to the region upstream of *adc* did not result in significant reporter levels in the presence of any *bfmRS* allele (Fig 5D), suggesting that the construct tested did not contain sufficient information for transcript initiation or stability.

We next tested whether BfmRS is capable of regulating a subset of these promoter-reporter fusions in *E*. *coli* (Fig 5C), which lacks highly similar orthologs of BfmRS. WT BfmRS stimulated the activity of the *slt*, *tolB*, *ompW* and ACX60_RS18040 promoters when introduced heterologously in *E*. *coli* (Fig 5E). Moreover, co-introduction of BfmR with an inactive BfmS variant in which a critical catalytic residue has been substituted (G494V [5]) resulted in robust activation of all four reporters (Fig 5E). These results are consistent with direct regulation of these target gene promoters by BfmRS. Reporter fusion to the *oxa51* promoter region showed little activity when co-expressed with WT or variant BfmRS in *E*. *coli* (Fig 5E), suggesting that transcription initiation from this region requires other elements present in *A*. *baumannii*. The ACX60_RS09685 promoter did not show robust stimulation by either WT or hyperactive BfmRS when heterologously expressed in *E*. *coli* (Fig 5E), reproducing its behavior in *A*. *baumannii*.

Multiple pathways related to envelope biogenesis and division showed coordinated transcriptional regulation by BfmRS. Therefore, the following analysis pursued the hypothesis that control of the cell envelope underlies the dramatic resistance phenotypes associated with altered BfmRS signaling.

### BfmRS regulation of β-lactamase production contributes to β-lactam resistance and occurs via noncanonical pathways

**Fig 6:**
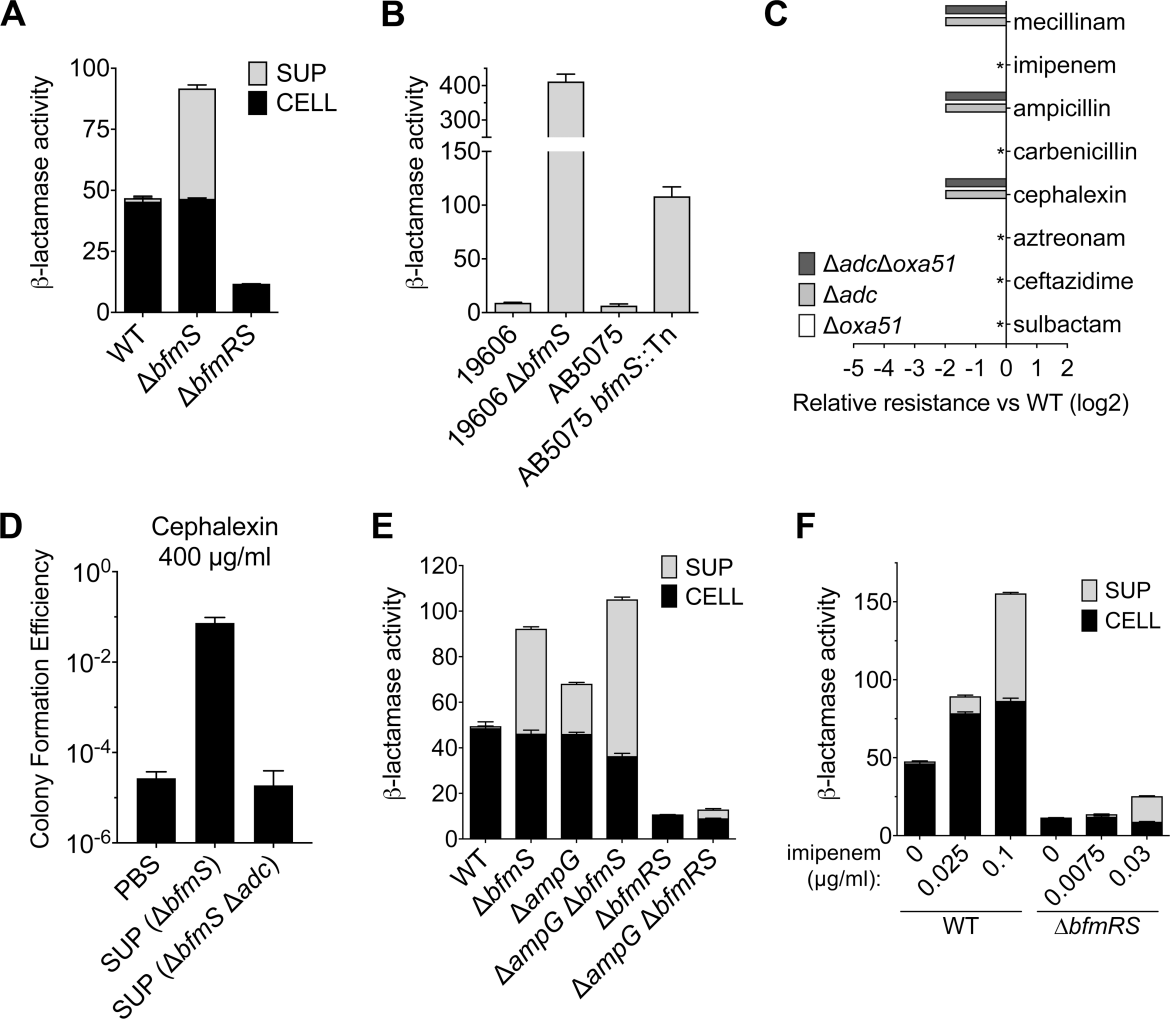
β-lactamase up-regulation by BfmRS occurs via noncanonical pathways. (A) β-lactamase is differentially regulated by BfmRS. *A*. *baumannii* 17978 WT or *bfmRS* mutants were grown to early post-exponential phase and β-lactamase activity (Vmax/A_600_) in cell sonicates (CELL) or culture supernatants (SUP) was quantified by using Nitrocefin as substrate. Bars show mean ± s.d. (n = 3). (B) Additional *A*. *baumannii* isolates (ATCC 19606 and AB5075, and their *bfmS* mutant counterparts) were grown to early post-exponential phase, and culture supernatants were assayed for β-lactamase activity as above. Bars show mean ± s.d. (n = 3). (C) *A*. *baumannii* 17978 β-lactamases confer resistance to a limited range of substrates. Relative resistance vs. WT of bacteria deleted for the indicated β-lactamase gene was determined as in Fig 2A. The Δ*oxa51* strain showed same MIC as WT for all drugs tested. For drugs marked with *, all three mutant strains showed same MIC as WT. (D) Extracellular β-lactamase provided *in trans* enhances ability of sensitive bacteria to grow with antibiotics. PBS or filtered cell-free supernatants (SUP) from cultures of the indicated strains were used as diluent for WT bacteria spotted on plates containing cephalexin at 400 μg/ml. Bars show geometric mean ± s.d. (n = 4). (E) β-lactamase augmentation by BfmRS occurs in the absence of the AmpG transporter. β-lactamase activity was measured after culture of the indicated mutants as in A. Bars show mean ± s.d. (n = 3). (F) BfmRS contributes to high-level β-lactamase induction by imipenem. Mid-log phase cultures were treated with imipenem for 2 hours before processing cell sonicates and supernatants for β-lactamase activity as in *A*. Bars show mean ± s.d. (n = 3).

To solidify the connection between BfmRS control of cell wall homeostasis and antibiotic resistance, we next analyzed β-lactamase modulation by BfmRS. Transcription of the two chromosomal genes encoding β-lactamases (*adc* and *oxa51*) was greatly increased in Δ*bfmS* and decreased in the Δ*bfmRS* mutant (Fig 5A). By using mutants with deletions of these genes, we found that the chromogenic substrate nitrocefin enabled detection of activity from the ADC β-lactamase specifically (S6A Fig), as noted previously [24]. WT *A*. *baumannii* bacteria produced a level of β-lactamase activity in cell sonicates that was decreased about 4-fold in Δ*bfmRS* (Fig 6A). The Δ*bfmS* strain had WT levels of cell-associated β-lactamase activity, but had greatly increased levels relative to WT secreted into culture supernatants (Fig 6A). Total cell protein in these supernatants was at or near the limit of detection, and at least 40-fold lower than that measured in cell sonicates (S6B Fig and Materials and Methods), arguing against cell lysis playing a major role in enhanced β-lactamase release by the Δ*bfmS* strain. Increased extracellular secretion of β-lactamase due to *bfmS* inactivation was observed with additional *A*. *baumannii* clinical isolates (ATCC 19606 and AB5075; Fig 6B). Deletion of the ADC β-lactamase resulted in increased sensitivity to mecillinam, ampicillin, and cephalexin, indicating the protein contributes to resistance to these antibiotics (Fig 6C, S6C Fig, and S4 Table). We found that the filtered supernatant fraction from Δ*bfmS* cultures allowed WT bacteria to form colonies at higher efficiency on plates containing an otherwise inhibitory concentration of cephalexin (Fig 6D). Enhanced resistance was due to ADC, because no growth enhancement was observed with culture supernatants from Δ*bfmS Δadc* double mutants (Fig 6D). These results demonstrate that β-lactamase production is modulated by BfmRS and enhances resistance to the appropriate antibiotics.

To understand further how BfmRS enhances β-lactamase production, we explored its relationship to the well-described β-lactamase control circuit, which depends on cell wall recycling by the AmpG permease and is activated by β-lactam-mediated cell wall damage [25–27]. Stimulation of β-lactamase production by hyperactive BfmR still occurred in the absence of the AmpG permease, consistent with regulation occurring independently of this cell wall recycling component (Fig 6E; Δ*ampG* Δ*bfmS*). This is in contrast to the prototypical muropeptide-sensing AmpR system, which requires AmpG permease for AmpC β-lactamase induction [28]. In fact, loss of AmpG increased the extracellular levels of β-lactamase in all strains, regardless of the BfmRS allele (Fig 6E; compare Δ*ampG* strains to isogenic controls), consistent with a lack of genetic interdependence between *ampG* and *bfmRS*. β-lactamase release in the absence of AmpG was not associated with wholesale release of cellular protein content, consistent with selective secretion rather than cell lysis (S6D Fig). These results demonstrate that β-lactamase control by BfmRS occurs independently of AmpG-promoted recycling, and reveal two parallel pathways for control of β-lactamase production.

To gain evidence that BfmRS participates in the response to β-lactam-induced cell wall damage, strains were exposed to graded increases of the β-lactam imipenem. At sub-MIC levels, there were corresponding increases in production of β-lactamase, including substantial release extracellularly (Fig 6F) without extensive release of cellular protein content (S6E Fig). Parallel exposure of Δ*bfmRS* cells to imipenem at doses causing a similar degree of growth inhibition resulted in a muted response (Fig 6F), although there was evidence for some induction of β-lactamase, consistent with a parallel BfmRS-independent response. These results support the model that *A*. *baumannii* β-lactamase production has diverged from canonical Gram-negative induction systems, requiring activation of multiple control pathways to maximize protection from β-lactams.

### β-lactamase-independent resistance to cell wall stress is linked to BfmRS control of cell division and lytic transglycosylase production

Based on several results, we argue that in addition to control of the chromosomal β-lactamases, BfmRS confers β-lactamase-independent resistance to cell wall damaging antibiotics. Lack of BfmRS resulted in hypersensitivity to many β-lactams (imipenem, carbenicillin, aztreonam, and ceftazidime) whose potency was not affected by WT levels of the chromosomal β-lactamases (compare Fig 2A with Fig 6C and S6C Fig). Furthermore, the degree of resistance conferred to antibiotics that were clear substrates of the β-lactamases (mecillinam, ampicillin, and cephalexin) could only partially account for the decreased overall resistance in the *bfmRS* null strain, in which β-lactamase production is decreased but not abolished (compare Figs 2A and 6C). Finally, deletion of *adc* had only minimal effect on the resistance of Δ*bfmS* to ceftazidime and aztreonam (S6C Fig; compare with Fig 2B), although a role for augmented OXA-51 levels in this enhanced resistance has not been examined.

We considered the possibility that BfmRS augmentation of capsular polysaccharide [5] contributes to β-lactam resistance. A multi-gene deletion involving the K locus (ΔKL3) that eliminates capsule production and causes a truncated lipoolosaccharide [5] did not substantially affect resistance levels against carbenicillin or aztreonam in a *bfmRS*^+^ strain or in a strain with a truncated (null) *bfmS* allele [5] (S7A Fig). The ΔKL3 deletion showed increased CFE with ceftazidime, particularly in the *bfmS*-null strain (S7A Fig). These findings argue against the model that BfmRS-mediated resistance to β-lactams derives from increased production of K-locus polysaccharides.

**Fig 7:**
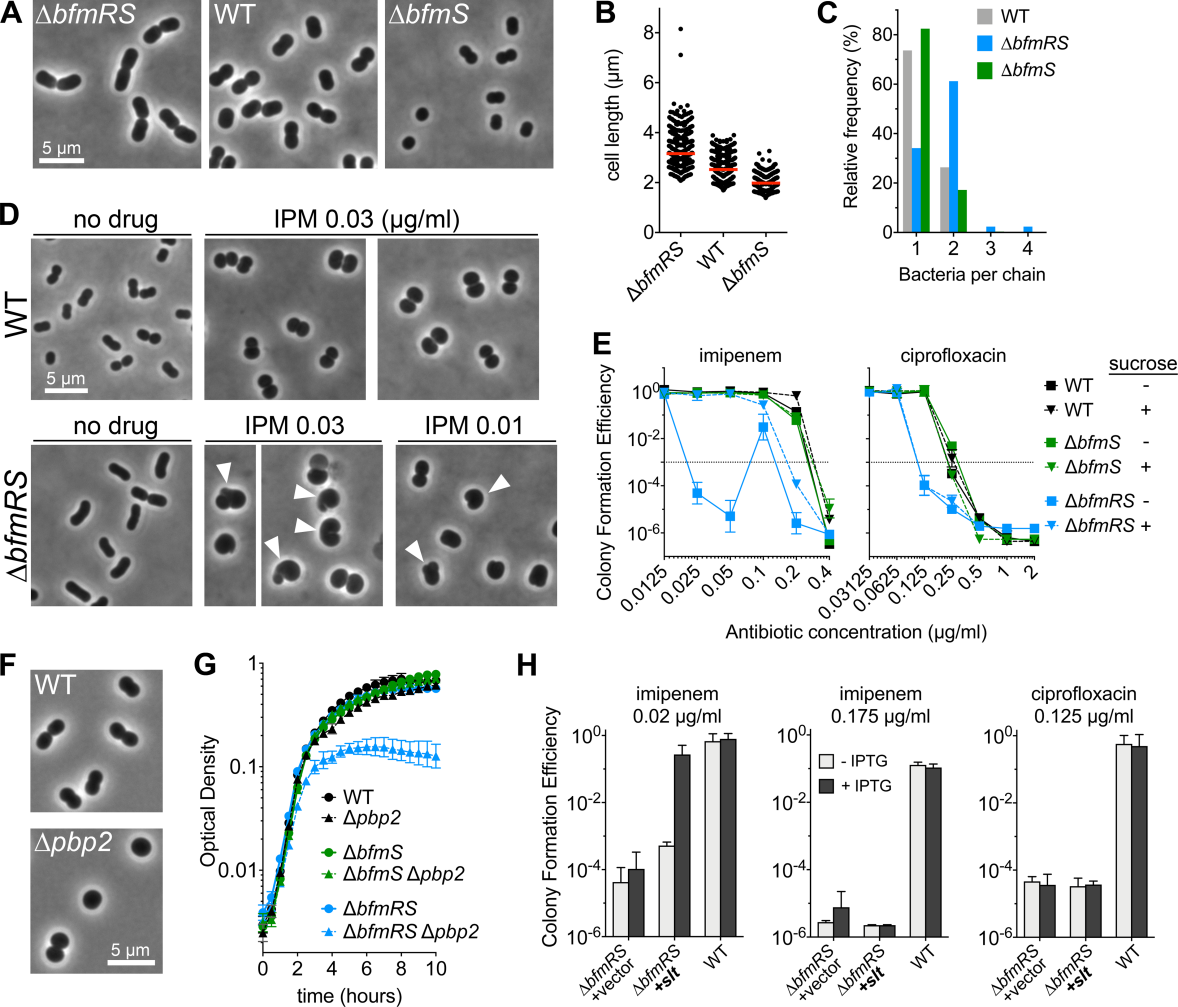
A strategy for BfmRS control of resistance to cell wall stress that is independent of its control of β-lactamase production. (A-C) BfmRS modulates cell morphogenesis. (A) Mid-log phase bacteria were imaged with phase contrast optics. (B) Cell length from n > 300 cells per strain was quantified; Red lines indicate median values. Mutants differed significantly from WT (P <0.0001, Mann-Whitney test). (C) Chaining was quantified for bacteria counted in panel *B*. Histogram shows frequency of chains containing the indicated number of cells. One chain of 5 cells and one chain of 10 cells were observed with Δ*bfmRS* but are not included in histogram. (D) Bacteria lacking *bfmRS* experience cell morphology defects (arrowheads) at low concentrations of imipenem (IPM). Mid-log phase cultures were treated with imipenem at the indicated concentration for 2 hours and bacteria were imaged as in *A*. (E) Selective suppression of the resistance defect of Δ*bfmRS* bacteria with 7.5% sucrose. Bacteria were grown on solid medium containing increasing concentrations of the indicated antibiotics, as well as 0 or 7.5% sucrose, and CFE determined. Data points show geometric mean ± s.d. (n = 3). (F) *pbp2* deletion causes loss of rod morphology and propagation as spheres. Bacteria were grown to log phase in antibiotic-free LB medium and imaged via phase-contrast microscopy. (G) *bfmRS* deletion amplifies growth defect caused by *pbp2* deletion. Bacterial growth was measured as change in optical density during 37°C incubation (Materials and Methods). Data points represent mean ± s.d. (n = 3). (H) Increasing *slt* expression partially suppresses imipenem hypersensitivity in cells lacking BfmRS. EGA495 (Δ*bfmRS*) harboring vector control (pEGE305) or derivative with *slt* gene downstream of T5-*lac*p promoter (pEGE316) were grown on solid medium containing the indicated antibiotic supplemented with or without IPTG at 1mM. Data points show geometric mean of CFE ± s.d. (n = 3). 17978 WT was included as additional control (n = 2).

We hypothesized that BfmRS control of peptidoglycan growth pathways that enhance cell division contributes to β-lactamase-independent resistance based on gene expression analysis (Figs 4 and 5). Consistent with this analysis and with a previous report [5, 9], we found that mutations in *bfmRS* had major effects on cell morphogenesis. Under conditions of exponential growth in LB medium in which WT bacteria grow as short rods (median length 2.5 μm), length was greatly increased with Δ*bfmRS* cells (3.2 μm), while Δ*bfmS* cells assumed a smaller, more spherical morphology having a diameter of 2.0 μm (Fig 7A and 7B; Materials and Methods). In addition, alterations in the degree of bacterial chaining were observed. Chains of two or more connected bacteria occurred at higher frequency in the Δ*bfmRS* strain compared to the WT and Δ*bfmS* strains (Fig 7C and S5 Fig). These changes indicate that BfmRS increases the frequency of division and cell separation.

To test the model that control of cell division and morphogenesis contributes to β-lactamase-independent resistance, we analyzed the effects of *bfmRS* mutation on *A*. *baumannii* morphology after challenge with imipenem. Sub-MIC levels of imipenem (0.03 μg/ml) caused bacteria with WT BfmRS to grow as single or diploid spheres (Fig 7D), a phenotype consistent with the known targeting of the cell wall elongation machinery by this antibiotic and related carbapenems [29, 30]. We reasoned that BfmRS facilitates the intrinsic ability to bypass imipenem-mediated elongation block by promoting growth and division as spheres. Consistent with this notion, at 0.03 μg/ml imipenem, a concentration at which growth is dependent on BfmRS (Fig 2B and S7B Fig), Δ*bfmRS* bacteria showed aberrant morphologies including larger, uneven, less spherical shapes that appeared to reflect division plane defects, including blebbing and incomplete pinching between connected spheroids (Fig 7D). Aberrant non-spherical morphology was also evident with a lower dose (0.01 μg/ml, Fig 7D) at which Δ*bfmRS* CFE is predicted to be 100% (Fig 2B). Furthermore, the loss of the regulatory system appeared associated with osmotic fragility, as the CFE of *bfmRS*-null cells on graded levels of imipenem approached WT levels in the presence of 7.5% sucrose (Fig 7E, left), albeit with smaller colonies than observed for the *bfmRS*^+^ control (S7C Fig). Consistent with an osmoprotectant function, sucrose did not significantly modify CFE of WT or Δ*bfmS* with imipenem, nor did it boost Δ*bfmRS* CFE on medium containing ciprofloxacin, which does not cause cell lysis (Fig 7E, right). Absence of BfmRS did not cause bacteria to become osmotically fragile when grown in medium lacking antibiotics (S7D Fig), indicating that in the absence of drug-induced stress, the sacculus of this strain provides sufficient protection from irreversible osmotic damage.

As an additional test of the model that BfmRS facilitates growth and division pathways bypassing elongation block, we determined the role of BfmRS in protecting from mutational disruption of cell elongation. We isolated mutants lacking the elongation-specific peptidoglycan transpeptidase, PBP2, which is the target of mecillinam and imipenem [29, 30], and determined the genetic interactions of *pbp2* mutations with *bfmRS* alleles. Single deletion of *pbp2* produced viable bacteria that grew as spheres, reflective of a block in elongation system function (Fig 7F), and caused slightly reduced growth in the post-exponential phase (Fig 7G, WT vs Δ*pbp2*). These phenotypes resembled those caused by *pbp2* knockout in *Pseudomonas aeruginosa* [31]. Notably, the Δ*bfmRS* deletion caused a marked exacerbation of the Δ*pbp2-*associated growth defect (Fig 7G, Δ*bfmRS* vs Δ*bfmRS* Δ*pbp2*), resulting in much lower yield during growth in liquid medium and small colonies on solid medium (S7E Fig). An aggravating genetic interaction was not observed with the constitutively active Δ*bfmS* allele (Fig 7G, Δ*bfmS* vs Δ*bfmS* Δ*pbp2*). Lack of BfmRS activity therefore strongly modulated the phenotype of the elongation-defective Δ*pbp2* mutant. The resilience of BfmRS-active cells challenged with varied elongation system lesions is consistent with the ability of BfmRS to permit cell propagation through enhanced division complex levels or activity.

In *E*. *coli*, cells become hypersensitive to β-lactam inhibition of elongation when insufficient division complex levels are combined with knockout of the key lytic transglycosylase enzyme Slt [19]. Slt activity protects cells by preventing toxic peptidoglycan misincorporation resulting in shape defects [19]. Because absence of BfmRS results in extremely low expression of *slt* (Fig 5) in the setting of reduced division complex function (Figs 5, 7A–7D, 7F and 7G), we hypothesized that restoring *slt* expression in Δ*bfmRS* bacteria would relieve toxicity due to elongation-targeting β-lactams. When *slt* expression is increased via induction from a multicopy plasmid, Δ*bfmRS* cells indeed show elevated resistance to low levels of imipenem (0.02μg/ml) (Fig 7H). Restoration of *slt* expression did not augment resistance at a higher concentration of imipenem (0.175μg/ml), or with ciprofloxacin (Fig 7H). With these conditions, killing is not predicted to be effectively suppressed by osmoprotection (Fig 7E). Together, these results are consistent with a model in which BfmRS counters β-lactam morphogenic toxicity through coordinate regulation of cell division and lytic transglycosylase activity.

### Bypass of antibiotic and serum hypersensitivity in mutants lacking *bfmRS* by mutations that restore envelope morphogenesis

**Fig 8:**
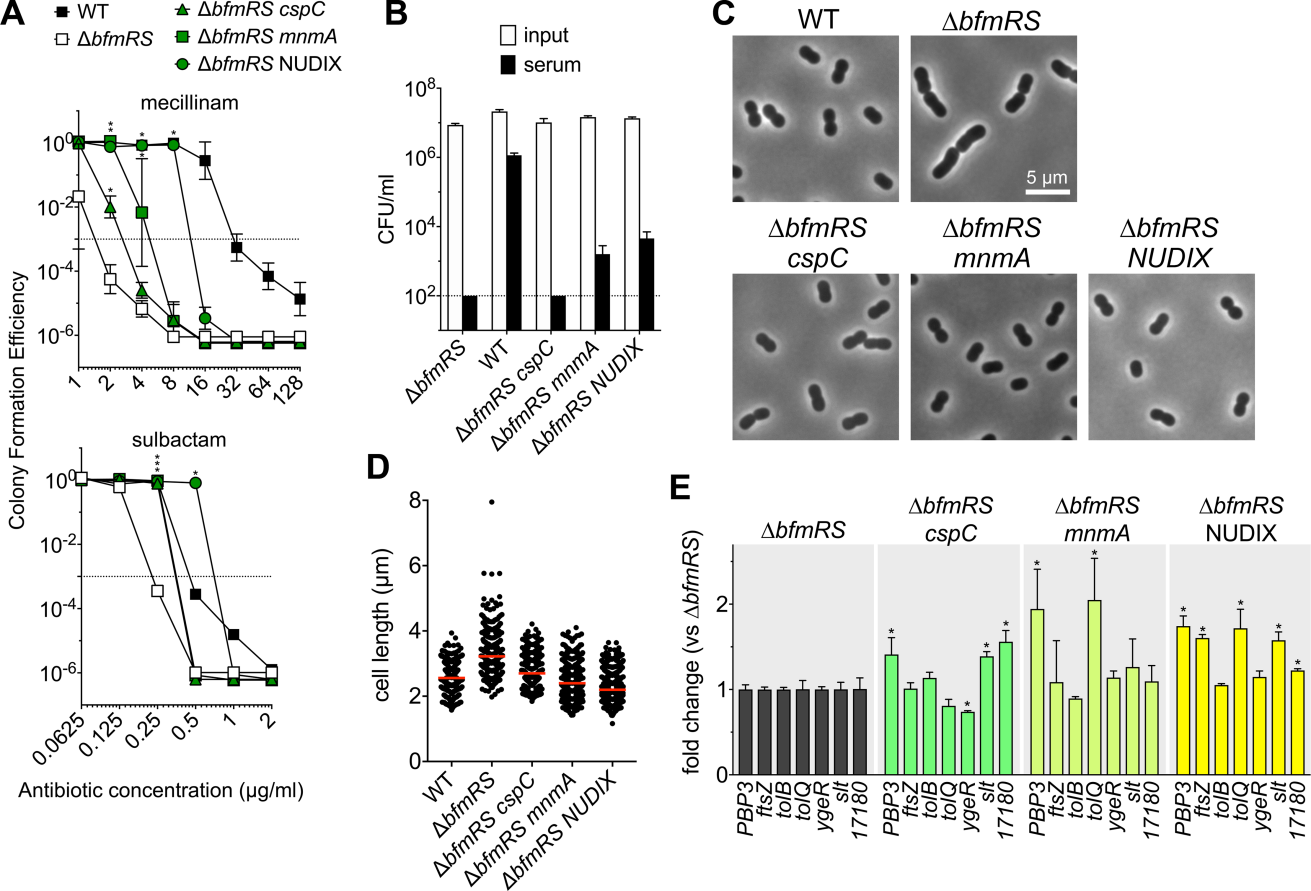
Hypersensitivity to antibiotics and serum killing of Δ*bfmRS* strain can be bypassed by mutations that affect control of cell morphogenesis. Spontaneous mutants derived from Δ*bfmRS* were isolated after selection with mecillinam or imipenem, and three isolates were analyzed for BfmRS-dependent phenotypes: EGA534 [Δ*bfmRS cspC*(G17A*fs*X21)], EGA555 [Δ*bfmRS mnmA*(G362S)], and EGA552 [Δ*bfmRS ACX60_RS05385/*(NUDIX)::*ISAba1*]. (A) Strains were tested for antibiotic resistance by CFE assay. Data points show geometric mean ± s.d. (mecillinam, n = 4; sulbactam, n = 2). *, P < 0.015 in unpaired t-tests comparing mutant vs WT; asterisks were stacked in the case of overlapping symbols. (B) Ability to withstand complement-mediated killing in serum was examined as in Fig 1. Lines show mean ± s.d. (n = 3). The limit of detection is indicated by the dotted line. (C) Cellular morphology of Δ*bfmRS* suppressor mutants. Bacteria were grown in antibiotic-free LB medium and imaged via phase-contrast microscopy. (D) Cell length was determined from n > 240 cells per strain. Red lines represent median values. Suppressor mutants differed significantly from Δ*bfmRS* (P < 0.0001, Mann-Whitney test). (E) Transcript levels were quantified via qPCR, and the fold change in each mutant vs the parental Δ*bfmRS* strain was determined. Bars show the mean value ± s.d. (n = 3). Bars marked with * had P < 0.05 (unpaired t-tests) and at least 20% difference in mean when comparing each mutant with parental Δ*bfmRS*.

The above findings indicate that BfmRS protection from cell wall stress is closely linked to control of division and morphogenesis. To determine processes in which intrinsic resistance and division/morphogenesis are co-regulated, we isolated suppressor mutations that bypass Δ*bfmRS* antibiotic hypersensitivity. Several spontaneous mutants of Δ*bfmRS* were isolated that could grow on plates containing otherwise inhibitory concentrations of mecillinam or imipenem [29, 30]. The resulting suppressor mutations were identified by whole-genome sequencing and were found to affect proteins having a range of functions (S5 Table). Several of the affected gene products participate in translation, while a particularly strong suppressor was identified in a gene encoding a predicted Nudix hydrolase (S5 Table; Fig 8). Mutations altering tRNA synthesis and Nudix hydrolases cause increased cellular levels of ppGpp in other bacterial species [32–35], with consequent increased resistance to mecillinam [34, 35]. Mutations in genes without an obvious connection to ppGpp levels or mecillinam resistance were also isolated. These included mutation in the putative genes *cspC* (cold-shock family RNA chaperone [36]), *pbp1a* (bifunctional peptidoglycan synthase [21]), *ctpA* (periplasmic protease [37]) and *ispB* (isoprenoid quinone synthesis [38]).

We analyzed the intrinsic resistance and cell morphology of a subset of these suppressors, which were determined by whole-genome sequencing to have a single mutation in *cspC* (frameshift), *mnmA* (tRNA modification enzyme, G362S substitution predicted to be deleterious), or ACX60_RS05385 (NUDIX hydrolase, gene disrupted by insertion sequence). These strains each showed increased CFE with mecillinam compared to the parental Δ*bfmRS* strain, with particularly robust suppression resulting from disruption of the NUDIX hydrolase (Fig 8A, top). Resistance to sulbactam, which targets cell division, was also augmented (Fig 8A, bottom), while in the case of *mnmA* and NUDIX mutations, survival in serum was enhanced (Fig 8B). The increased resistance phenotype in these strains was linked to restoration of a shorter rod shape comparable to WT (Fig 8C and 8D). With other suppressors, cell morphology was either not dramatically different from the parent (*pbp1a* transpeptidase domain mutant, and *ispB* catalytic domain mutant) or resulted in irregularly shaped, thin rods (*ctpA* transposon insertional mutant) (S8A Fig). In suppressors showing restoration of a shorter, WT-like rod shape, transcription of multiple cell division and peptidoglycan degradation genes was also increased relative to the Δ*bfmRS* parent, indicating that they have effects on levels of bacterial transcription of a large swath of genes associated with the cell envelope (Fig 8E). The phenotypes resulting from mutations in these strains link increased intrinsic resistance with proper control of cell division, phenocopying major functions of BfmRS.

## Discussion

Virulence and antibiotic resistance are intertwined in *A*. *baumannii* [2, 6, 39, 40]. In this work we demonstrated that the pathogen employs BfmRS to coordinate both its ability to cause disease and to resist a broad range of antibiotics. We revealed that BfmRS functions to globally influence the transcriptome. Expression of genes involved in biogenesis and growth of multiple envelope components (in addition to capsule [5]) was highly dependent on the state of BfmRS signaling, pointing to transcriptional control of envelope homoeostasis as a major mechanism of resistance to various forms of antimicrobial attack. With the cell-wall targeting β-lactam antibiotics, BfmRS controls resistance through two strategies: β-lactamase production and physiological resistance to cell wall lesions. Our results indicate that BfmRS co-regulates the envelope biogenesis and division machineries, connecting its control of pathogenicity and drug resistance.

This study demonstrates that a single genetic locus can control conversion of *A*. *baumannii* to a state of increased pathogenicity. A transition to increased virulence is driven by growth on sub-MIC levels of the translation inhibitor chloramphenicol [5], and translation inhibition appears connected to BfmRS signaling. Full induction of capsular polysaccharides by chloramphenicol treatment requires BfmRS [5], and several mutations suppressing *bfmRS*-null phenotypes isolated in this work are predicted to affect translation. Although our suppressor analysis has pointed to candidates that may act as transmitters of translation-level stress, such as mutations that affect the levels of ppGpp and cold shock proteins (discussed further below), the precise signaling network connecting BfmRS with ribosomal stress is unclear. In addition, the end-targets under transcriptional regulation that mediate control of virulence remain to be elucidated. The breadth of the BfmRS modulon suggests that many factors are likely involved. In dissecting how this regulatory network functions in disease processes, an important approach would be to expand the use of animal models including those that directly examine bacteremia, pneumonia, and other forms of *A*. *baumannii* disease.

The BfmRS system controls broad-range intrinsic antibiotic resistance in addition to determining pathogenicity. BfmRS-deficient mutants showed hypersensitivity to many antibiotics with a diverse range of targets, indicating that the system maintains the function of broadly protective intrinsic resistance factors. Transcriptome profiling pointed to multifaceted coordination of cell envelope processes which potentially bolster the envelope permeability barrier. This is supported by the low-level hypersensitivity of Δ*bfmRS* to hydrophobic antibiotics such as rifampicin and erythromycin, although the presumed envelope defects were not sufficient to cause altered susceptibility to the large peptide antibiotic vancomycin (Fig 2A). BfmRS control of interactions with antibiotics appears to have multiple layers, as different *bfmRS* alleles caused impressive changes in resistance against β-lactams antibiotics, as well as altered persistence in assays after transient exposure to high concentrations of diverse antibiotics.

We uncovered that BfmRS coordinates multiple defenses against cell wall lesions that can be roughly grouped into β-lactamase-dependent and -independent pathways. With the β-lactamase-dependent pathway, resistance is conferred against a subset of β-lactams through a non-canonical regulatory strategy that does not depend on traditional AmpG-mediated cell wall recycling. We also provide evidence that BfmRS controls a β-lactamase-independent resistance strategy, linked to the ability to counter toxicity induced by β-lactams. Our data support a model in which BfmRS maintains the integrity of the peptidoglycan sacculus in the face of β-lactam stress by directly controlling cell division and cell wall degradation pathways (in which Slt plays a major role), preventing accumulation of toxic peptidoglycan structures. In addition, BfmRS may allow cells to counteract depletion of cell wall precursors, which is another aspect of β-lactam intoxication [19]. BfmRS increases transcription of many genes involved in production of precursors of the cell wall and other envelope structures. One key precursor is Und-P, a lipid carrier for intermediates in the biosynthesis of envelope carbohydrate polymers including peptidoglycan and capsule [41]. Increased Und-P levels may limit β-lactam toxicity and may also account for the ability of *bfmS* mutation to suppress lethality due to capsule assembly block [5], a type of lesion that depletes Und-P due to sequestration into dead-end intermediates [42].

We isolated suppressor mutants that bypassed the β-lactam hypersensitivity caused by *bfmRS* deletion, to identify processes that may couple resistance to control of cell morphogenesis and possibly collaborate with BfmRS signaling. One set of suppressors included a cold shock family protein (Csp). Csps form a large family of RNA-binding proteins that regulate gene expression [36] which can either be induced in response to low temperature or produced constitutively at 37°C [43]. Interestingly, some Csps are induced by treatment with chloramphenicol, a stress that also stimulates capsule overproduction and transition to a hypervirulent state in *A*. *baumannii* [5]. The *A*. *baumannii csp* gene that was affected in our suppressor analysis was very highly expressed during growth at 37°C (S1–S3 Datasets), indicating that it probably has important functions at a variety of temperatures. An additional large group of suppressors of Δ*bfmRS* hypersensitivity had mutations predicted to slow translation. Many of these mutations are predicted to result in lower cellular aminoacyl tRNA levels, which is known to elevate ppGpp levels and activate the stringent response [35]. This response increases mecillinam resistance in *E*. *coli* for unknown reasons, although enhancements to peptidoglycan precursor pools or cell division capacity, and reductions in overall peptidoglycan synthesis and turnover, have been proposed as possible mechanisms [44–46]. Interestingly, BfmRS modulates the expression of several of the tRNA synthesis or modification genes that had suppressor mutations (S8B Fig), consistent with feedback interactions in which control of translation rates interplays with envelope morphogenesis and division.

We propose that the *Acinetobacter* BfmRS signaling cascade enhances physiological properties that are known in other Gram-negatives to be activated upon entry into the post-exponential phase of growth [47]. This model is supported by multiple findings: BfmRS increased tolerance to bactericidal antibiotic exposures, drove the construction of a cellular morphology that had reduced size relative to strains lacking the circuit, enhanced growth yield in post-exponential phase, and controlled the expression of post-exponential phase-associated genes known to protect against oxidative and osmotic stresses and redirect metabolism [47]. BfmRS is also known to mediate biofilm formation [9], a lifestyle associated with post-exponential phase transition or starvation in *Acinetobacter* [48].

In *E*. *coli* and related Gram-negative bacteria, transcriptional reprogramming during transition to post-exponential phase is governed by the alternative sigma factor σ^S^ (RpoS), whose levels and/or activity are augmented by a histidine-kinase signaling system (Rcs) [49]. *Acinetobacter* does not encode an ortholog of RpoS, or of the several components of the Rcs phosphorelay system, indicating that responses to starvation and other stresses are occurring via noncanonical pathways. Given the similarities between phenotypes of null and hyperactive alleles of BfmRS and Rcs, including antibiotic resistance [50], capsule biosynthesis [5], and biofilm formation [9, 51], BfmRS may represent a functional counterpart to the Rcs phosphorelay system. It is almost certain that BfmRS, like Rcs, acts both directly on target gene promoters as well as indirectly through additional regulators to exert control on its expansive modulon. Direct control of targets modulating the envelope (including those identified in Fig 5E) could have pleiotropic effects accounting for many indirect changes in gene expression. Based on the multiple cell envelope processes under BfmRS control, and the contribution of BfmRS to imipenem-induced β-lactamase production, envelope-level stress represents a potential input that is sensed by this system.

Understanding the global control network in which BfmRS plays a key role will illuminate how *A*. *baumannii* optimizes its ability to grow under stress conditions and transitions to states of augmented virulence and resistance. Targeting this system therapeutically represents an attractive strategy to enhance the effectiveness of antibiotic treatments while inhibiting pathogenesis.

## Materials and methods

### Bacterial strains, growth conditions, and antibiotics

The bacterial strains used in this study are listed in S1 Table. *A*. *baumannii* strains were derivatives of ATCC 17978 unless otherwise noted. Bacteria were grown in Lysogeny Broth (LB) (10 g/L tryptone, 5 g/L yeast extract, 10 g/L NaCl). Gentamicin (Gm, 15 μg/ml), kanamycin (Km, 10 μg/ml), carbenicillin (Cb, 100 μg/ml), and/or tetracycline (Tc, 10 μg/ml) (Sigma) were used at noted concentration in solid medium. Broth cultures were grown at 37°C in flasks with orbital shaking at 250 rpm or in tubes with rotation on a roller drum at 56 rpm. Growth was monitored by measuring absorbance spectrophotometrically at 600nm (A_600_).

### Molecular cloning and mutant construction

Plasmids and oligonucleotides (Integrated DNA Technologies) used in this study are listed in S1 and S2 Tables, respectively. All constructs were sequenced (Genewiz) before introduction into *A*. *baumannii* via electroporation. Δ*bfmRS*::*aacC1* and Δ*bfmR*::*aacC1* mutants were isolated via homologous recombination with previously described pSR47S (Km^R^)-based constructs [5], using methods that minimized exposure to antibiotics during strain construction. Km^S^ recombinant deletion mutants were isolated from Gm^R^ Km^R^ merodiploids by sucrose counterselection on medium lacking Gm. Markerless, in-frame deletions of additional genes were isolated via homologous recombination as described previously [5]. To engineer altered *bfmRS* alleles for complementation tests, inverse PCR was performed on fragments cloned in pUC18. The *bfmR*^*+*^*ΔbfmS* allele was generated with primers that encoded a 3X-FLAG tag fusion to the C-terminus of BfmR. The *bfmR*^*+*^*bfmS*(H234Q) allele was generated by using primers containing the engineered point mutation and a template encoding a 3X-FLAG tag fusion to the C-terminus of BfmS. Constructs were subcloned to pEGE148 and introduced in single copy at the Δ*bfmRS*::*aacC1* locus via homologous recombination. Chromosomal alleles of *bfmRS* were also cloned in pJB1801 for expression in *E*. *coli*.

A vector for transcriptional fusions to GFP, pEGE245, was constructed by replacing the EcoRI-PstI fragment of pWH1266 with a synthetic oligonucleotide containing tandem *rrnB*T1 & T7Te terminators and a SacI restriction site, followed by incorporation of the SacI-PstI fragment of pFPV25 (gift from Raphael Valdivia, Addgene plasmid # 20667) containing *gfp*mut3. Promoter fragments upstream of the indicated genes were cloned into pEGE245 directly upstream of the promoterless *gfp*mut3 gene. A vector for inducible expression, pEGE305, was constructed by replacing the EcoRI-PstI fragment of pWH1266 with a polylinker, followed by the *lacI*^*q*^ T5-*lac*p fragment cloned from pCA24N-*dinB* (gift of Veronica Godoy). The *slt* gene was cloned directly downstream of the T5-*lac*p promoter in pEGE305.

### Animal experiments

8–10 week old female C57BL/6 mice were used for intraperitoneal infections. WT and Δ*bfmS A*. *baumannii* were grown in LB to early post-exponential phase. Systemic infections were initiated by intraperitoneal injection of approximately 10^8^ bacteria suspended in 100ul PBS as previously described [5]. Animals showing signs of severe morbidity were euthanized by CO_2_ asphyxiation followed by cervical dislocation.

### Ethics statement

Experiments were carried out in accordance with protocols approved by Tufts University Institutional Animal Care and Use Committee (B2014-91) and in adherence with the Guide for the Care and Use of Laboratory Animals of the National Institutes of Health.

### Serum bactericidal assays

Approximately 10^5^ bacteria grown to early post-exponential phase were diluted in PBS and incubated with 60% baby rabbit complement serum (AbD Serotec) for one hour at 37°C. Serum inactivated by heating (56°C for 30 minutes) was used as a control. Reactions were stopped by placing on ice, and viable bacterial counts were determined by plating serial dilutions on LB agar followed by overnight incubation at 37°C.

### Analysis of antibiotic resistance and tolerance

Antibiotic resistance was measured by CFE assays utilizing solid medium containing 2-fold serial dilutions of antibiotic as previously described [5]. The lowest antibiotic concentration at which CFE is less than 10^−3^ defines the MIC. MIC values were used to calculate relative resistance levels of mutant strains compared to WT. For assays measuring bacterial viability after transient treatment with antibiotics, bacteria were grown to early post-exponential phase (A_600_ ~1.5–2), antibiotic was added, and cultures continued growth for 6 hours, with samples taken at the indicated time points. Optical density of samples was measured as A_600_, and the density of viable bacteria (colony forming units (CFU)/ml) was determined by washing samples with PBS, serially diluting in PBS, and plating onto solid medium lacking antibiotics. Bacteria surviving ciprofloxacin tolerance assays were tested for mutational resistance by passaging on LB agar without antibiotics, then assessing growth relative to naïve parental bacteria on solid medium containing 0.5 and 1 μg/ml ciprofloxacin.

### Microscopy

Mid-log phase bacteria were immobilized in agarose pads (1% in PBS), and images were acquired via phase-contrast on a Zeiss Axiovert 200m microscope with 100x/1.3 lens. Length of individual cells and chain number was measured by the Oufti software package [52].

### RNA-seq and enrichment analysis

#### RNA-seq

Three replicate cultures of WT and each *bfmRS* mutant strain were grown to A_600_ = 0.5. Cultures were combined with one volume of ice-cold acetone:ethanol (1:1) and frozen at -80°C until further processing. Cells were washed with TE and RNA was extracted via RNeasy (Qiagen). Removal of contaminating genomic DNA was achieved via two rounds of treatment with TURBO DNase (Ambion) and verified by PCR with 16S-specific primers as previously described [53]. rRNA was depleted by using Ribo-Zero Gram-Negative Bacteria kit (Illumina) and purified with Agencourt RNAClean XP beads (Beckman). Fragmentation, cDNA synthesis and library amplification were performed via the TruSeq Stranded Total RNA kit (Illumina) by the Tufts University Genomics Core Facility (TUCF-Genomics). Libraries were sequenced (single-end 50bp) on a HiSeq2500 with High Output V4 chemistry at TUCF-Genomics.

Reads corresponding to residual rRNA sequences were removed *in silico*, allowing for normalization of samples based on total number of reads representing non-rRNA transcripts and controlling for differences in rRNA depletion across samples, analogous to the previously described “RPKMO” approach [54]. Remaining genomic reads were aligned to the *A*. *baumannii* 17978-mff chromosome (NZ_CP012004) and plasmids (NC_009083, NC_009084, and NZ_CP012005) by using TopHat version 2.1.0, and transcriptome quantification and comparison across samples was performed via Cuffdiff version 2.2.1 [55]. Tests for differential gene expression used a q value (P value adjusted for multiple testing via false discovery rate procedure) threshold of 0.05. Venn diagrams were generated via BioVenn [56] and Venny [57].

#### Pathway enrichment analysis

Gene ontology (GO) terms for *A*. *baumannii* 17978 genes were assigned from matching or homologous entries in the Swiss-Prot database, and were counted across datasets via custom Perl scripts (available upon request). For biological process GO terms, an enrichment score was calculated as previously described [43]. Terms with an enrichment score of at least 2 and that were defined by at least 2 genes in the differentially expressed gene subset were subsequently analyzed for significance by using a hypergeometric test with Benjamini-Hochberg correction for multiple testing. Terms with a q value < 0.05 were considered significantly enriched.

### qPCR gene expression analysis

Bacteria were cultured and harvested as for RNA-seq. RNA was purified and cDNA synthesized as described previously [5]. cDNA was amplified with Power SYBR Green Master Mix (Applied Biosystems) via a StepOnePlus system according to the manufacturer’s instructions for two-step RT-PCR. Target amplification efficiency was assessed by generating a standard curve with a dilution series of cDNA and was determined to be >95% for each primer pair. Controls lacking reverse-transcriptase were performed to confirm lack of signal from residual genomic DNA. Gene expression levels were quantified by using the 2^-ΔΔCt^ method with two endogenous controls (16S and *rpoC*).

### GFP reporter assays

Bacteria containing derivatives of pEGE245 were grown to early post-exponential phase in LB without antibiotics (*A*. *baumannii*) or LB with carbenicillin and tetracycline at 20 and 3.5 μg/ml, respectively (*E*. *coli* with pJB1801::*bfmRS*). Cultures were transferred to 96-well plates (Costar 3631) and diluted with PBS. Fluorescence (excitation at 480 nm/emission at 520 nm) and A_600_ were measured with a Biotek SynergyMx plate reader. Fluorescence units (RFU) were normalized by dividing by A_600_, and RFU/A_600_ of parallel-cultured control bacteria containing the vector (pEGE245) alone was subtracted to calculate background-corrected reporter fluorescence values.

### β-lactamase assays

Bacteria were grown to early post-exponential phase, centrifuged, and cell pellets and supernatants collected. Cells were resuspended in ice-cold 0.1 M phosphate buffer (pH 7) and periplasmic contents liberated by sonication for 2 min (10 s on, 5 s off duty cycle) with a high-intensity cuphorn sonifier (Branson) chilled to 4°C. Samples were clarified by centrifugation, and extracts were diluted 5–10 fold in 0.1M phosphate buffer (pH 7). β-lactamase content was assayed spectrophotometrically with the colorimetric substrate nitrocefin (20μg/ml in 0.1M phosphate buffer, pH 7) by measuring absorbance at 486 nm every minute for 15 minutes at room temperature. β-lactamase activity was calculated as initial reaction rate (Vmax)/culture density (A_600_)*dilution factor. Supernatant samples were assayed as above either directly or after dilution in phosphate buffer. In antibiotic treatment experiments, mid-log phase cultures (A_600_ ~0.2) were treated with imipenem for 2 hours and processed as above. Total protein concentration was measured via Bradford microtiter assay (Bio-Rad) using BSA as standard.

### Osmotic down-shift assays

Bacteria were grown to mid-logarithmic or early post-exponential phase and then diluted 100-fold into ultrapure water. After incubation for 20-30minutes, 1/10^th^ volume of 10X PBS was added, and bacteria were serially diluted with 1X PBS and spotted onto LB agar plates for enumeration of surviving CFU.

### Whole-genome sequencing

Genomic library preparation for Illumina sequencing was performed using the small-volume Nextera tagmentation method [58], with the following modifications: NEBNext High-Fidelity 2X PCR Master Mix (NEB) was used for amplification; adapter addition/library amplification was followed by 5 cycles of reconditioning PCR with adapter-specific primers; library QC/quantitation was performed directly after PCR by visualizing a sample of each library separated on a 2% agarose/TAE gel; multiplexed PCRs were purified by using a single Qiagen PCR purification column and size-selected via a PippinHT. Libraries were sequenced (single-end 100bp or 150bp) on a HiSeq2500 at TUCF-Genomics. Reads were aligned to the *A*. *baumannii* 17978-mff genome and variants identified by using Geneious software [59] or BRESEQ [60]. The predicted functional impact of substitution variants was determined by using PROVEAN [61].

## Supporting information

S1 Fig BfmRS mutant growth in rich medium. Optical density was monitored during bacterial growth at 37°C in a Tecan M200 Pro plate-reader. Data points show mean ± s.d. (n = 3).
(PDF)

S2 Fig CFE assay data. (A) CFE data from which MICs (S3 Table) and relative resistance levels (Fig 2A) were calculated. MIC is defined as the antibiotic concentration at which CFE drops below 10^−3^ (dotted line). (B) Carbenicillin CFE assay testing Δ*bfmRS* strains with *bfmRS* alleles reintroduced in single copy. Data points show the geometric mean ± s.d. (n ≥ 2).
(PDF)

S3 Fig RNAseq reveals global changes in gene transcription in *bfmRS* mutants. (A) Venn diagrams showing differentially expressed genes present on plasmids (pAB1, pAB2, and pAB3) identified by pairwise comparison of RNAseq data between each mutant and WT. A q value < 0.05 was required to define differential expression for each comparison. (B) Fold change (log2) of every gene (mutant vs WT) was plotted along its genomic coordinate, showing concerted changes in transcript levels of plasmid-encoded genes. y-axis labels indicate every 200^th^ gene starting from origin of chromosome or plasmid.
(PDF)

S4 Fig Additional gene expression alterations dependent on BfmRS function. Gene ontology (GO)-term enrichment analysis of genes differentially expressed due to *bfmRS* mutations, showing remaining relationships not presented in Fig 4B. The Venn diagram is identical to that shown in Fig 4B and enriched GO biological process terms were identified as in Fig 4B. Δ*bfmS*-down and Δ*bfmS*-down Δ*bfmRS-*up had no significantly enriched terms.
(PDF)

S5 Fig Example images showing analysis of cell length and chaining. Phase-contrast images (left) were analyzed with the Oufti software package, utilizing default parameters for rod-shaped cells. Cell contours used for length calculation were defined (right). Cells constituted a chain if two cell contours (as defined by the image analysis) were connected at their poles.
(PDF)

S6 Fig β-lactamase production contributes to *A*. *baumannii* strain 17978 resistance to a limited range of β-lactam antibiotics. (A) The chromogenic substrate nitrocefin reports on the activity of the ADC β-lactamase. Deletion of *adc* but not *oxa51* causes loss of detectable nitrocefin hydrolysis. β-lactamase activity (Vmax/A_600_) was quantified from cell sonicates as in Fig 6A (n = 3). (B) Total protein concentration in samples analyzed in Fig 6A measured by Bradford assay. Protein concentration was not normalized for culture density. Bars show mean ± s.d. (n = 3). (C) CFE assays demonstrate that *A*. *baumannii* 17978 β-lactamases confer resistance to a limited range of substrates. Data points show geometric mean ± s.d. (n = 3). The Δ*bfmS* Δ*adc* strain was tested only in assays with ceftazidime and aztreonam. (D, E) Total protein concentration in samples analyzed in Fig 6E and 6F were determined by Bradford assay as in panel *B*.
(PDF)

S7 Fig Phenotypes relevant to β-lactamase-independent resistance in bacteria with altered BfmRS activity. (A) CFE assays with 17978 WT, EGA127 (*bfmS*^1-467^), EGA68 (ΔKL3), and EGA187 (*bfmS*^1-467^, ΔKL3). Data points show geometric mean ± s.d. (n = 3). (B) CFE was determined with imipenem 0.03 μg/ml. Bars show geometric mean ± s.d. (n = 3). (C) Images of colonies grown on plates from Fig 7E. IPM, imipenem. (D) Bacterial viability after osmotic down-shift. Bacteria were grown to the indicated A_600_ before down-shift by dilution into water. Bars show mean ± s.d. (n = 3). (E) Deletion of *pbp2* and *bfmRS* results in small colony phenotype. Colonies were imaged after overnight growth on LB agar plates.
(PDF)

S8 Fig Morphology phenotypes of suppressors of Δ*bfmRS* antibiotic hypersensitivity and BfmRS-dependent changes in expression of genes affected by suppressor mutation. (A) Bacteria were grown in antibiotic-free LB medium and imaged via phase-contrast microscopy. Suppressor mutants analyzed were: EGA564 [Δ*bfmRS ispB*(E93K)], EGA593 [Δ*bfmRS pbp1A*(G728S)], and EGA595 [Δ*bfmRS ctpA*::*ISAba11*]. EGA595 (Δ*bfmRS ctpA*::*ISAba11*) showed heterogeneous morphologies that were narrower than parental Δ*bfmRS*, with frequent irregularly shaped cells observed. (B) BfmRS-mediated alterations in expression of suppressor genes. Heat maps show log_2_ fold-change in transcription of each gene in mutant vs WT as determined by RNAseq. *, change is significant (q < 0.05).
(PDF)

S1 Table Strains and plasmids used in this study. (PDF)

S2 Table Oligonucleotide primers used in this study. (PDF)

S3 Table Minimal inhibitory concentrations (μg/ml) determined from colony formation efficiency assays testing *bfmRS* mutants. (PDF)

S4 Table Minimal inhibitory concentrations (μg/ml) determined from colony formation efficiency assays testing β-lactamase mutants. (PDF)

S5 Table Derivatives of Δ*bfmRS* containing mutations allowing suppression of antibiotic hypersensitivity. (PDF)

S1 Dataset RNA-seq data comparing Δ*bfmRS* to WT. (XLSX)

S2 Dataset RNA-seq data comparing Δ*bfmR* to WT. (XLSX)

S3 Dataset RNA-seq data comparing Δ*bfmS* to WT. (XLSX)
