# Supplementary figures and images for: A global regulatory system links virulence and antibiotic resistance to envelope homeostasis in Acinetobacter baumannii
Source: PLoS Pathog. 2018 May 24;14(5):e1007030. doi: 10.1371/journal.ppat.1007030 (PMC5967708; doi:10.1371/journal.ppat.1007030)

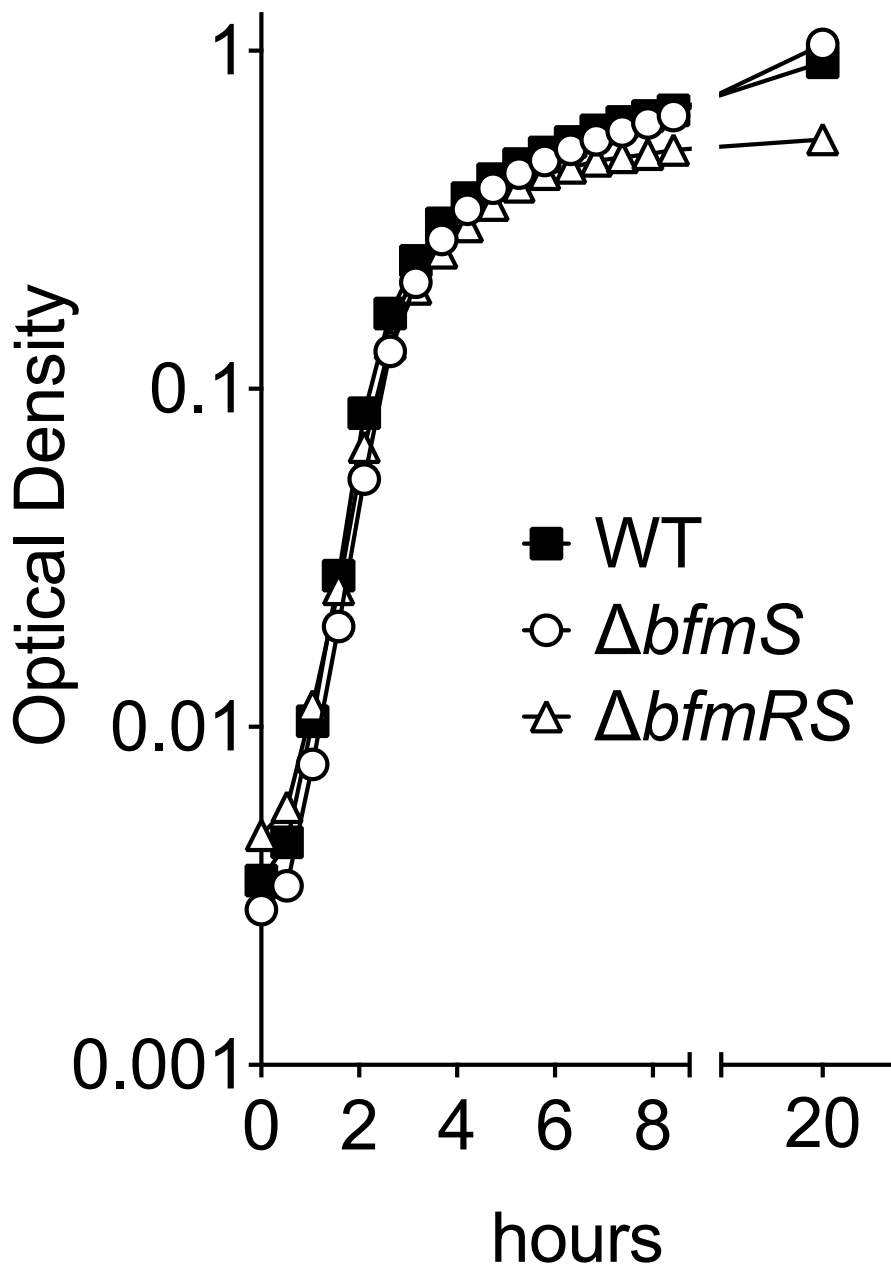

Supplement: S1 Fig — Optical density was monitored during bacterial growth at 37°C in a Tecan M200 Pro plate-reader. Data points show mean ± s.d. (n = 3). (PDF) [file ppat.1007030.s001.pdf]

**A**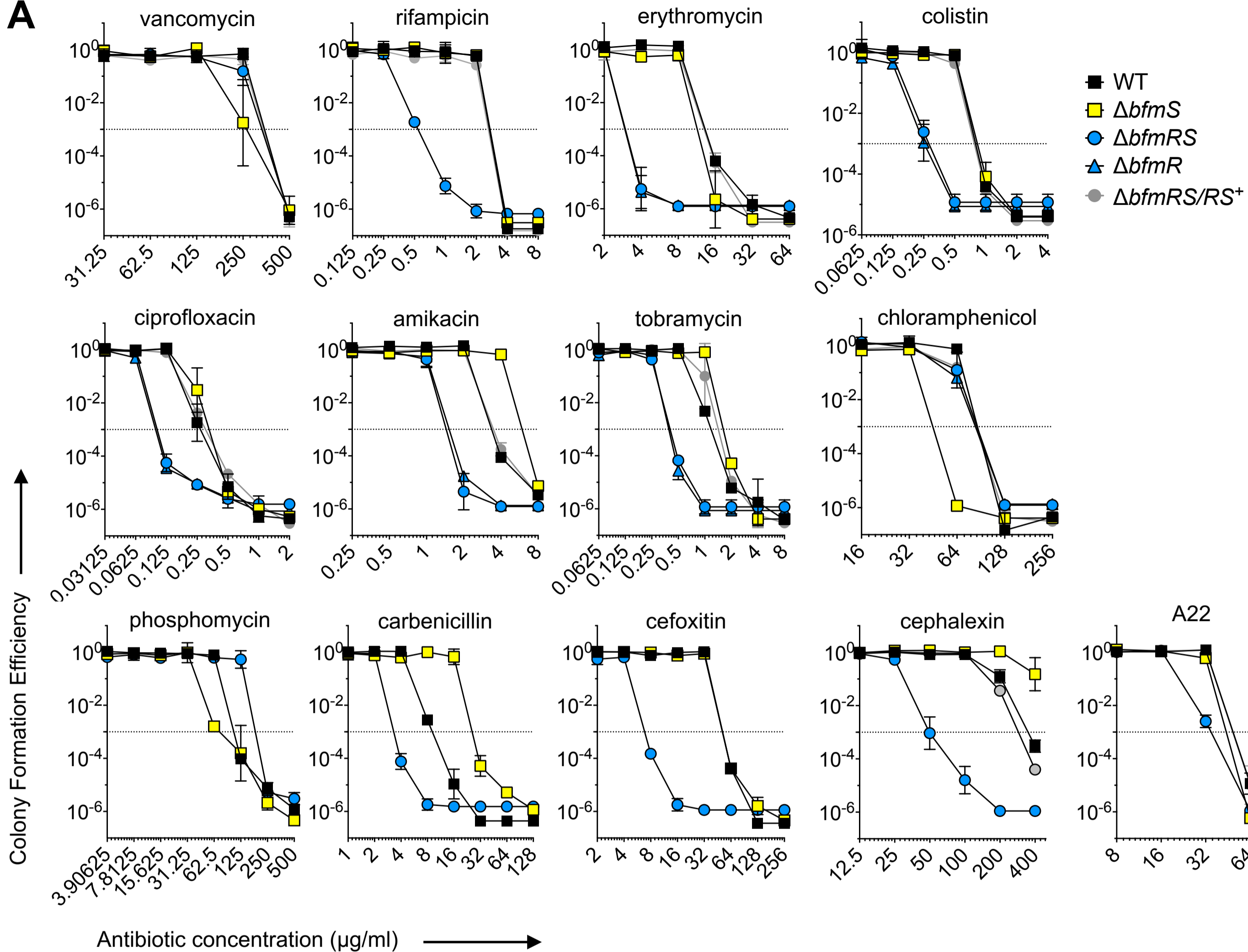**B**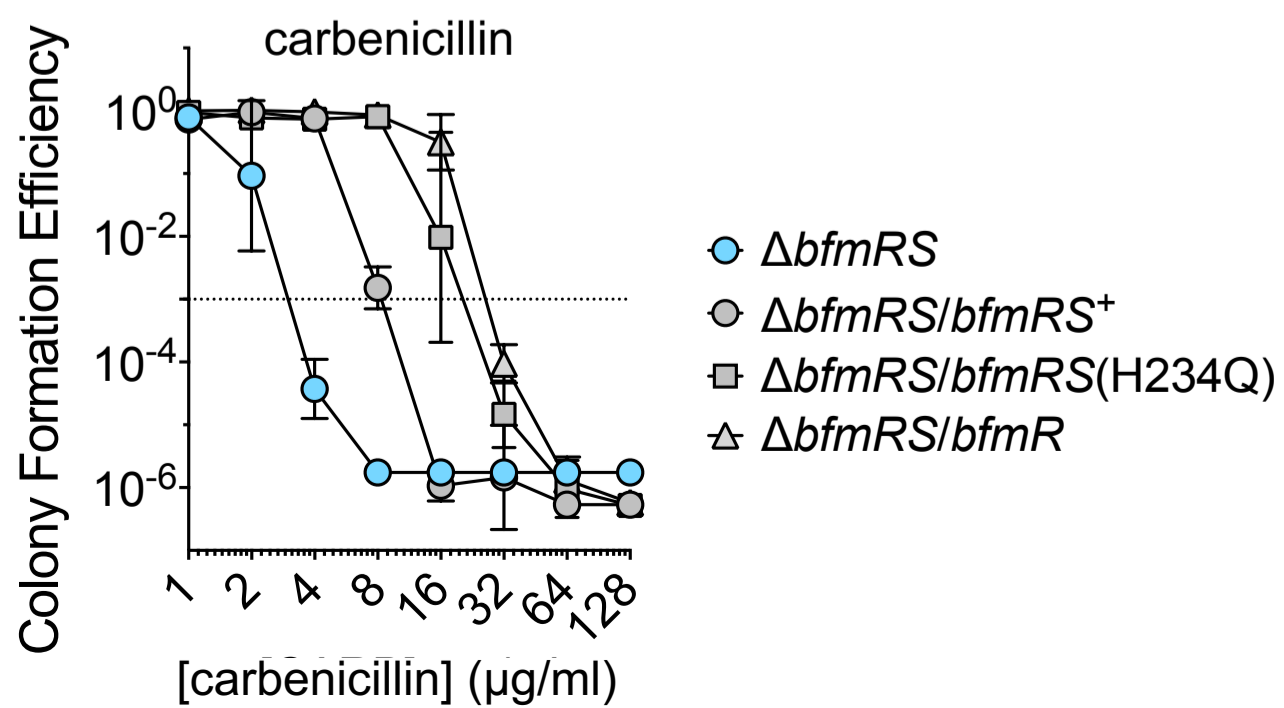

Supplement: S2 Fig — (A) CFE data from which MICs (S3 Table) and relative resistance levels (Fig 2A) were calculated. MIC is defined as the antibiotic concentration at which CFE drops below 10−3 (dotted line). (B) Carbenicillin CFE assay testing ΔbfmRS strains with bfmRS alleles reintroduced in single copy. Data points show the geometric mean ± s.d. (n ≥ 2). (PDF) [file ppat.1007030.s002.pdf]

**A**

174 total plasmid genes

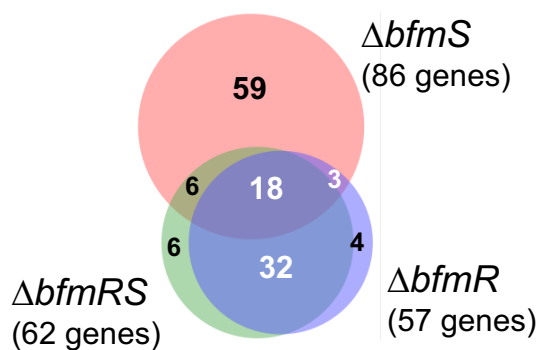**B**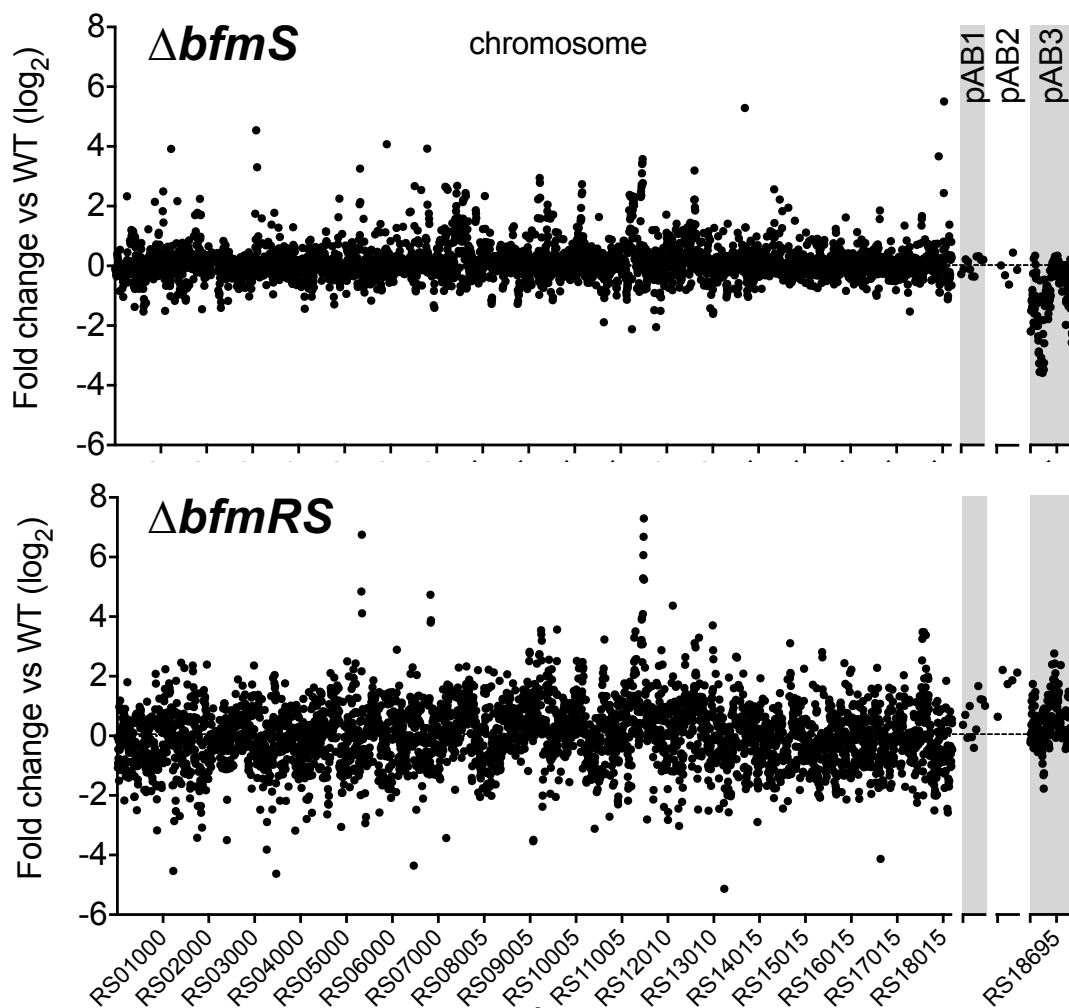

Supplement: S3 Fig — (A) Venn diagrams showing differentially expressed genes present on plasmids (pAB1, pAB2, and pAB3) identified by pairwise comparison of RNAseq data between each mutant and WT. A q value < 0.05 was required to define differential expression for each comparison. (B) Fold change (log2) of every gene (mutant vs WT) was plotted along its genomic coordinate, showing concerted changes in transcript levels of plasmid-encoded genes. y-axis labels indicate every 200th gene starting from origin of chromosome or plasmid. (PDF) [file ppat.1007030.s003.pdf]

WT

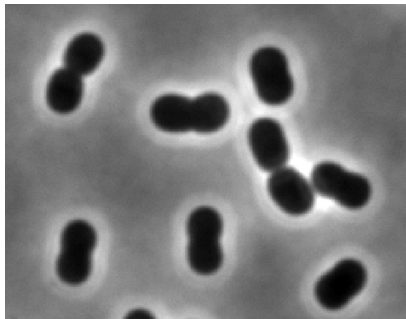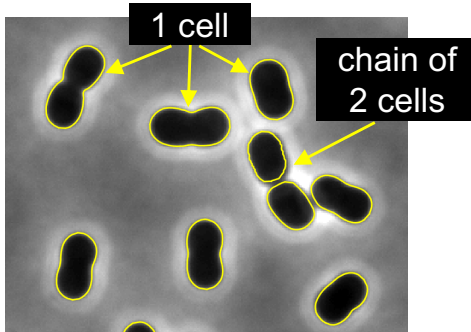

$\Delta bfmRS$

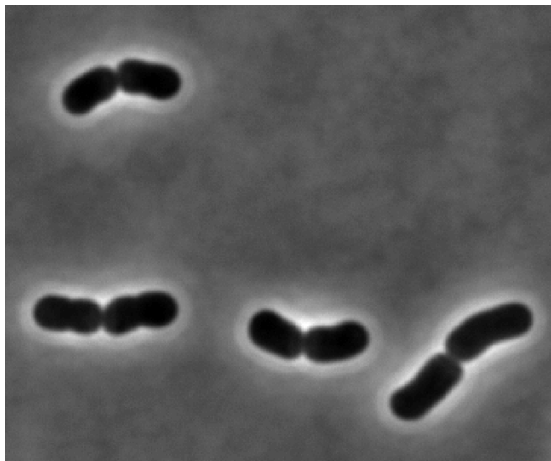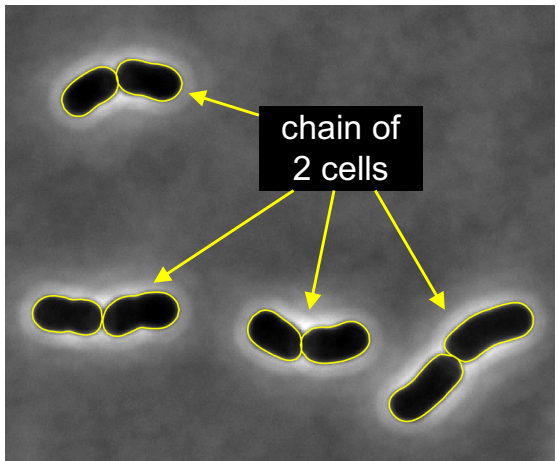

Supplement: S5 Fig — Phase-contrast images (left) were analyzed with the Oufti software package, utilizing default parameters for rod-shaped cells. Cell contours used for length calculation were defined (right). Cells constituted a chain if two cell contours (as defined by the image analysis) were connected at their poles. (PDF) [file ppat.1007030.s005.pdf]

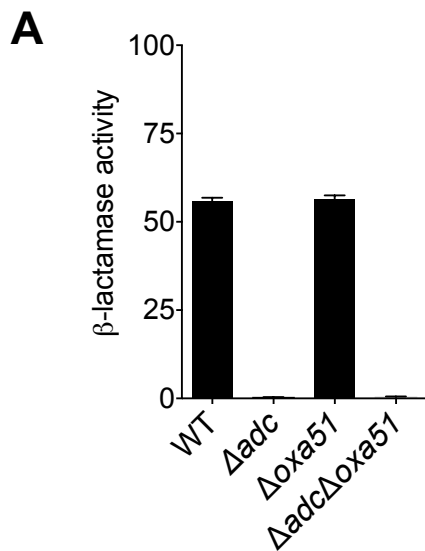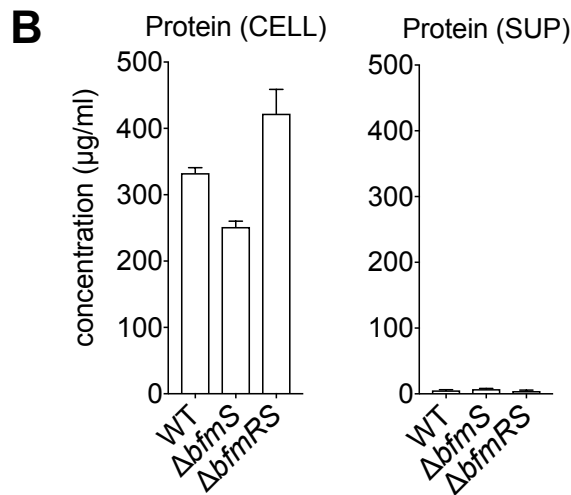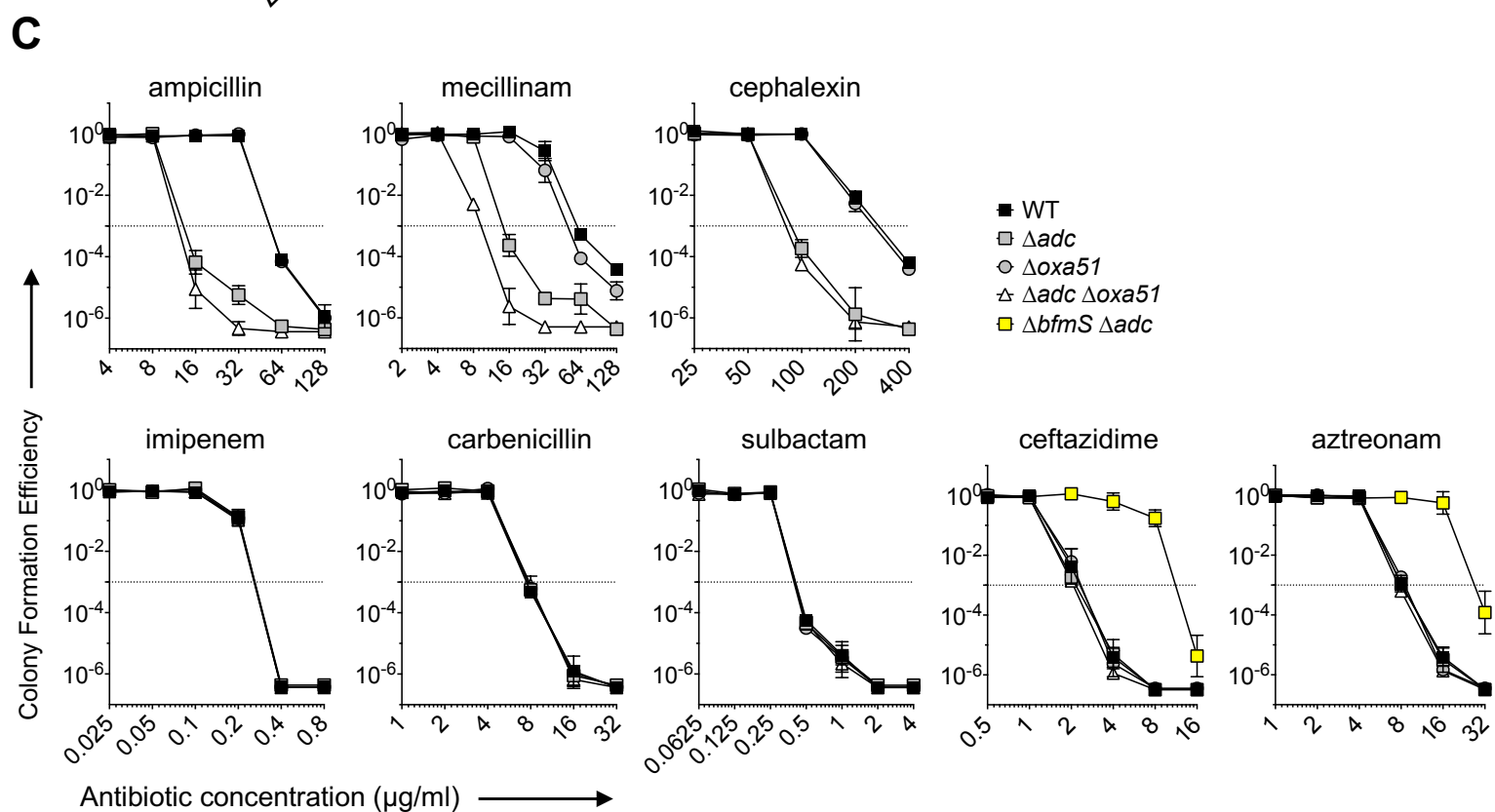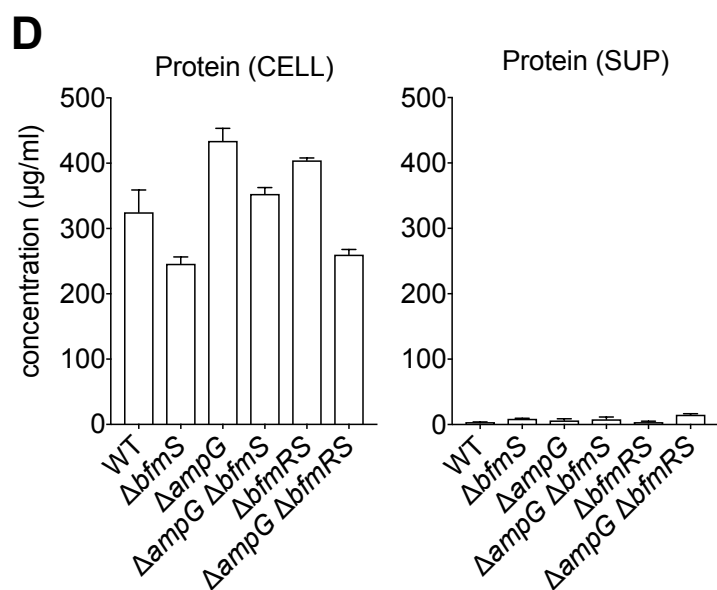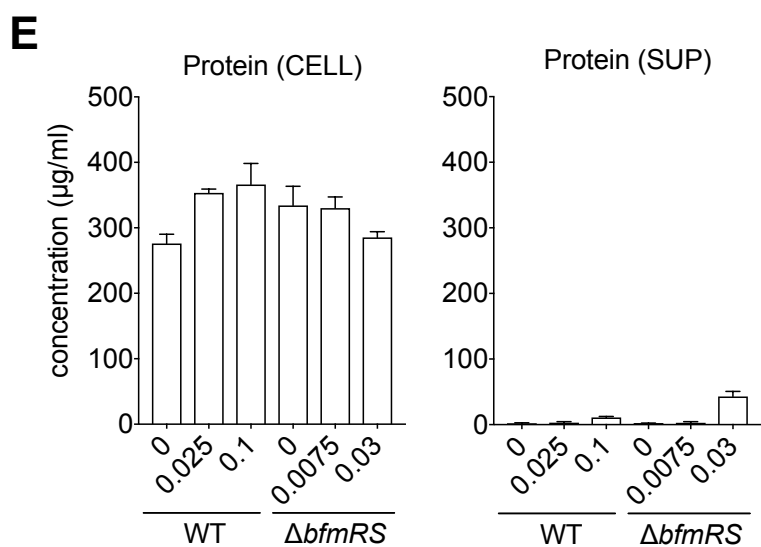

Supplement: S6 Fig — (A) The chromogenic substrate nitrocefin reports on the activity of the ADC β-lactamase. Deletion of adc but not oxa51 causes loss of detectable nitrocefin hydrolysis. β-lactamase activity (Vmax/A600) was quantified from cell sonicates as in Fig 6A (n = 3). (B) Total protein concentration in samples analyzed in Fig 6A measured by Bradford assay. Protein concentration was not normalized for culture density. Bars show mean ± s.d. (n = 3). (C) CFE assays demonstrate that A. baumannii 17978 β-lactamases confer resistance to a limited range of substrates. Data points show geometric mean ± s.d. (n = 3). The ΔbfmS Δadc strain was tested only in assays with ceftazidime and aztreonam. (D, E) Total protein concentration in samples analyzed in Fig 6E and 6F were determined by Bradford assay as in panel B. (PDF) [file ppat.1007030.s006.pdf]

**A**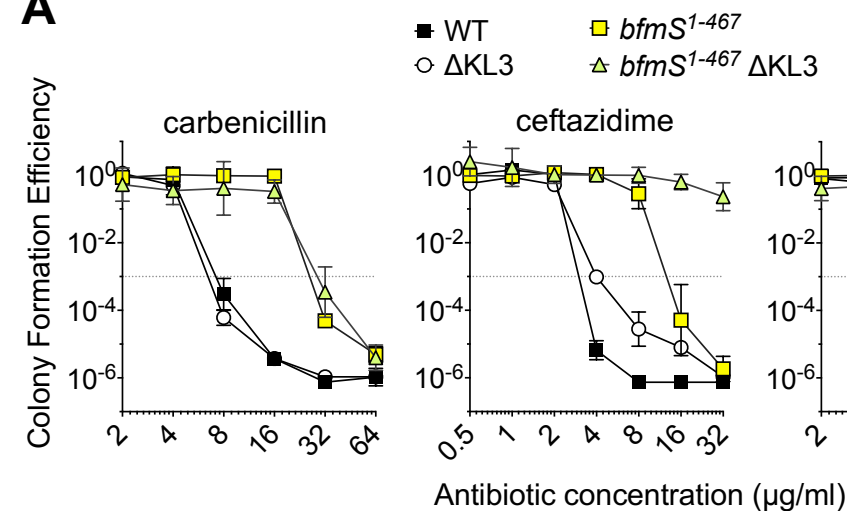**B**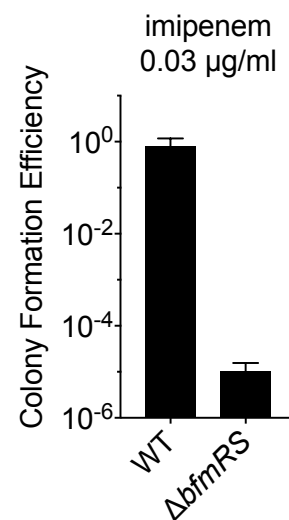**C**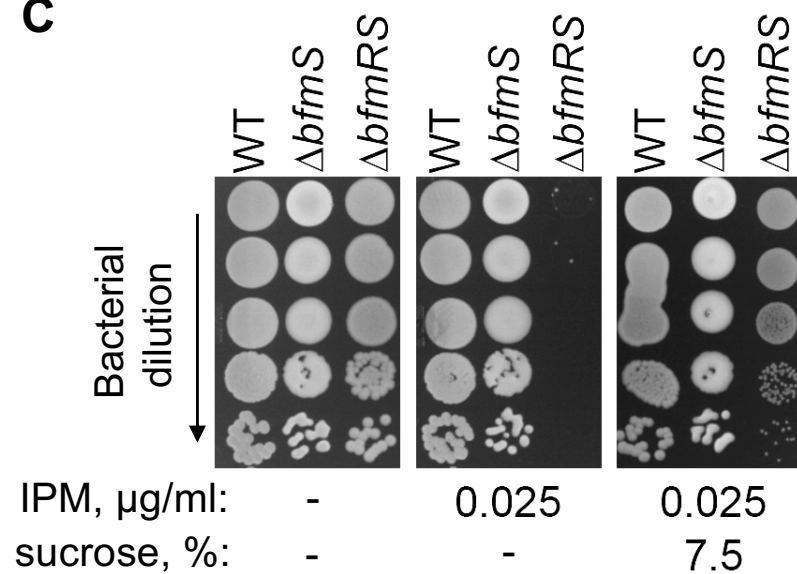**D**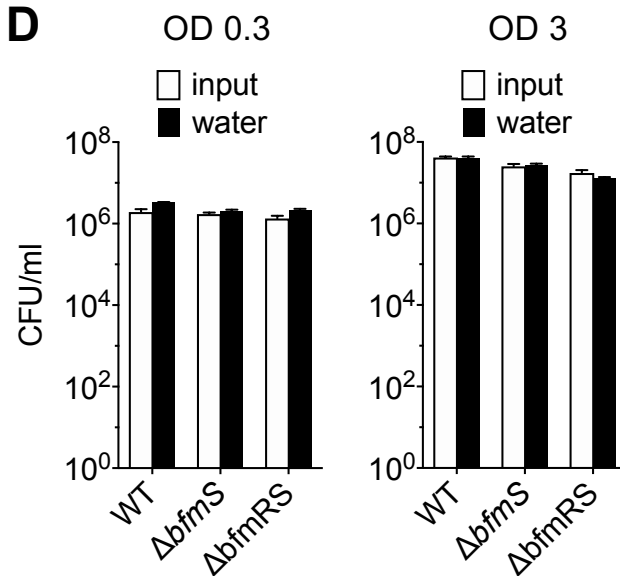**E**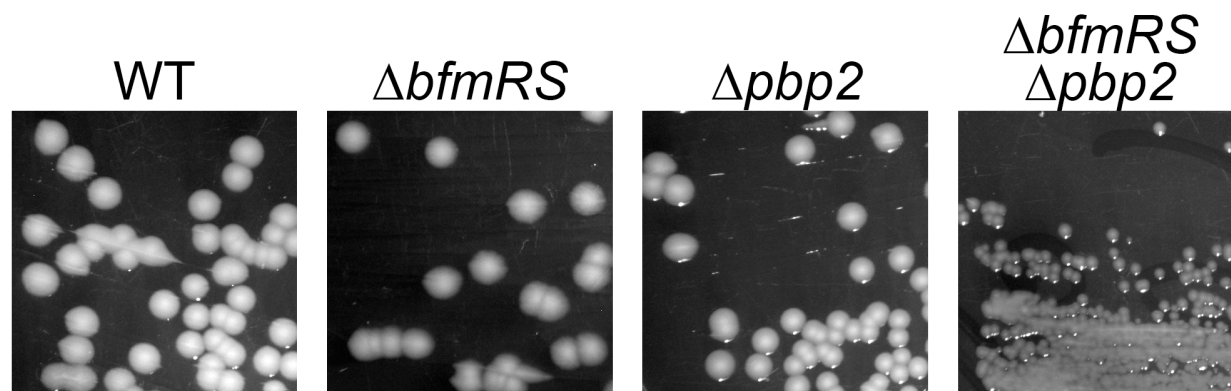

Supplement: S7 Fig — (A) CFE assays with 17978 WT, EGA127 (bfmS1-467), EGA68 (ΔKL3), and EGA187 (bfmS1-467, ΔKL3). Data points show geometric mean ± s.d. (n = 3). (B) CFE was determined with imipenem 0.03 μg/ml. Bars show geometric mean ± s.d. (n = 3). (C) Images of colonies grown on plates from Fig 7E. IPM, imipenem. (D) Bacterial viability after osmotic down-shift. Bacteria were grown to the indicated A600 before down-shift by dilution into water. Bars show mean ± s.d. (n = 3). (E) Deletion of pbp2 and bfmRS results in small colony phenotype. Colonies were imaged after overnight growth on LB agar plates. (PDF) [file ppat.1007030.s007.pdf]

**A**

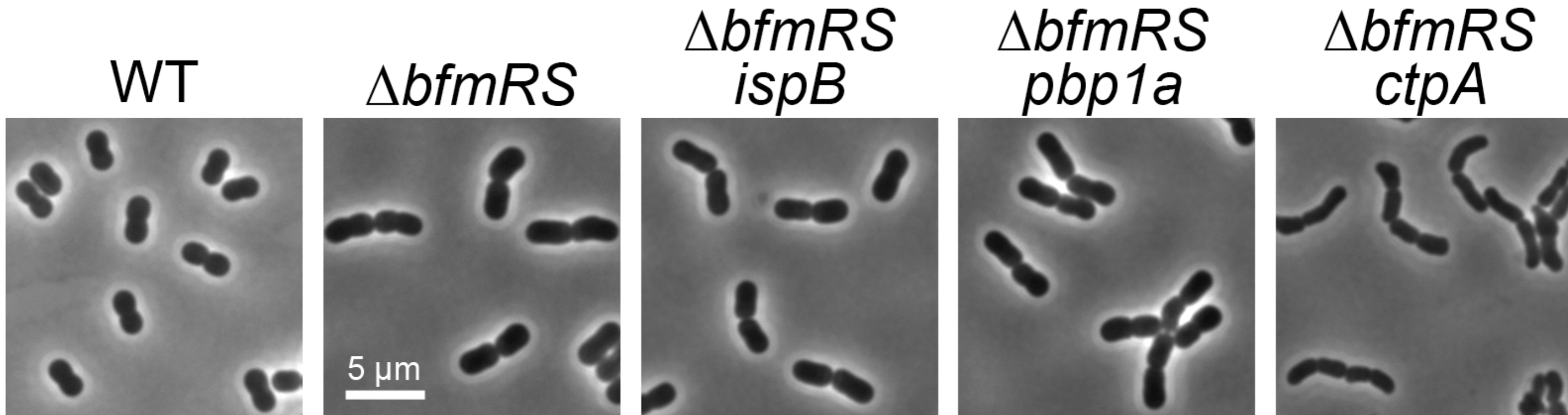

**B**

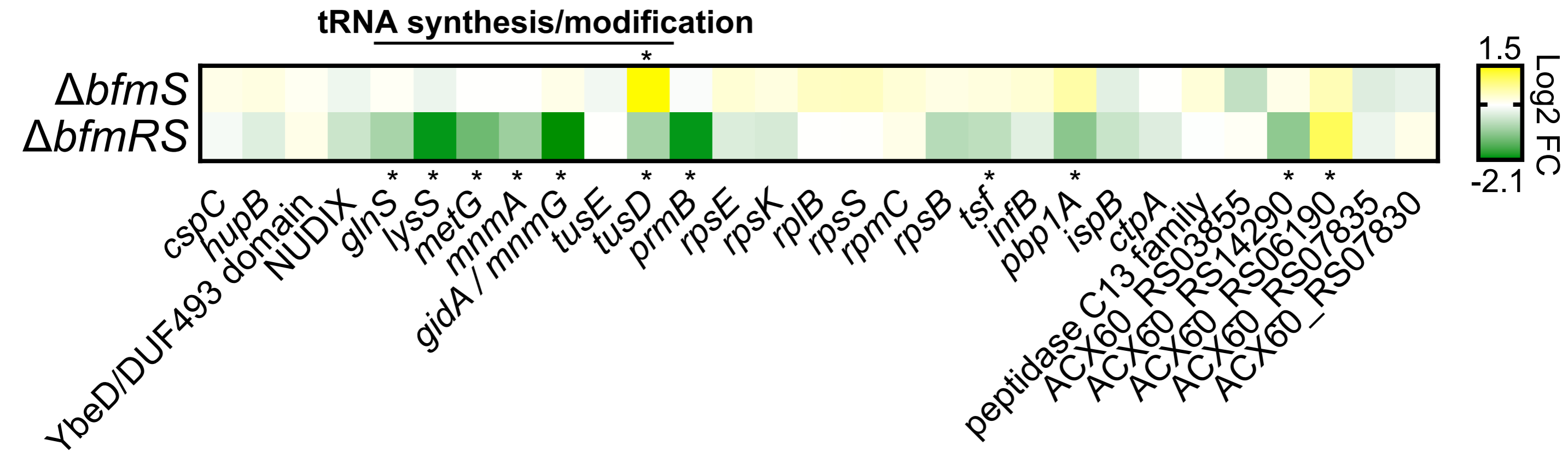

Supplement: S8 Fig — (A) Bacteria were grown in antibiotic-free LB medium and imaged via phase-contrast microscopy. Suppressor mutants analyzed were: EGA564 [ΔbfmRS ispB(E93K)], EGA593 [ΔbfmRS pbp1A(G728S)], and EGA595 [ΔbfmRS ctpA::ISAba11]. EGA595 (ΔbfmRS ctpA::ISAba11) showed heterogeneous morphologies that were narrower than parental ΔbfmRS, with frequent irregularly shaped cells observed. (B) BfmRS-mediated alterations in expression of suppressor genes. Heat maps show log2 fold-change in transcription of each gene in mutant vs WT as determined by RNAseq. *, change is significant (q < 0.05). (PDF) [file ppat.1007030.s008.pdf]
