# Supplementary material for: A global regulatory system links virulence and antibiotic resistance to envelope homeostasis in Acinetobacter baumannii
Source: PLoS Pathog. 2018 May 24;14(5):e1007030. doi: 10.1371/journal.ppat.1007030 (PMC5967708; doi:10.1371/journal.ppat.1007030)
Supplement: S4 Fig — Gene ontology (GO)-term enrichment analysis of genes differentially expressed due to bfmRS mutations, showing remaining relationships not presented in Fig 4B. The Venn diagram is identical to that shown in Fig 4B and enriched GO biological process terms were identified as in Fig 4B. ΔbfmS-down and ΔbfmS-down ΔbfmRS-up had no significantly enriched terms. (PDF) [file ppat.1007030.s004.pdf]

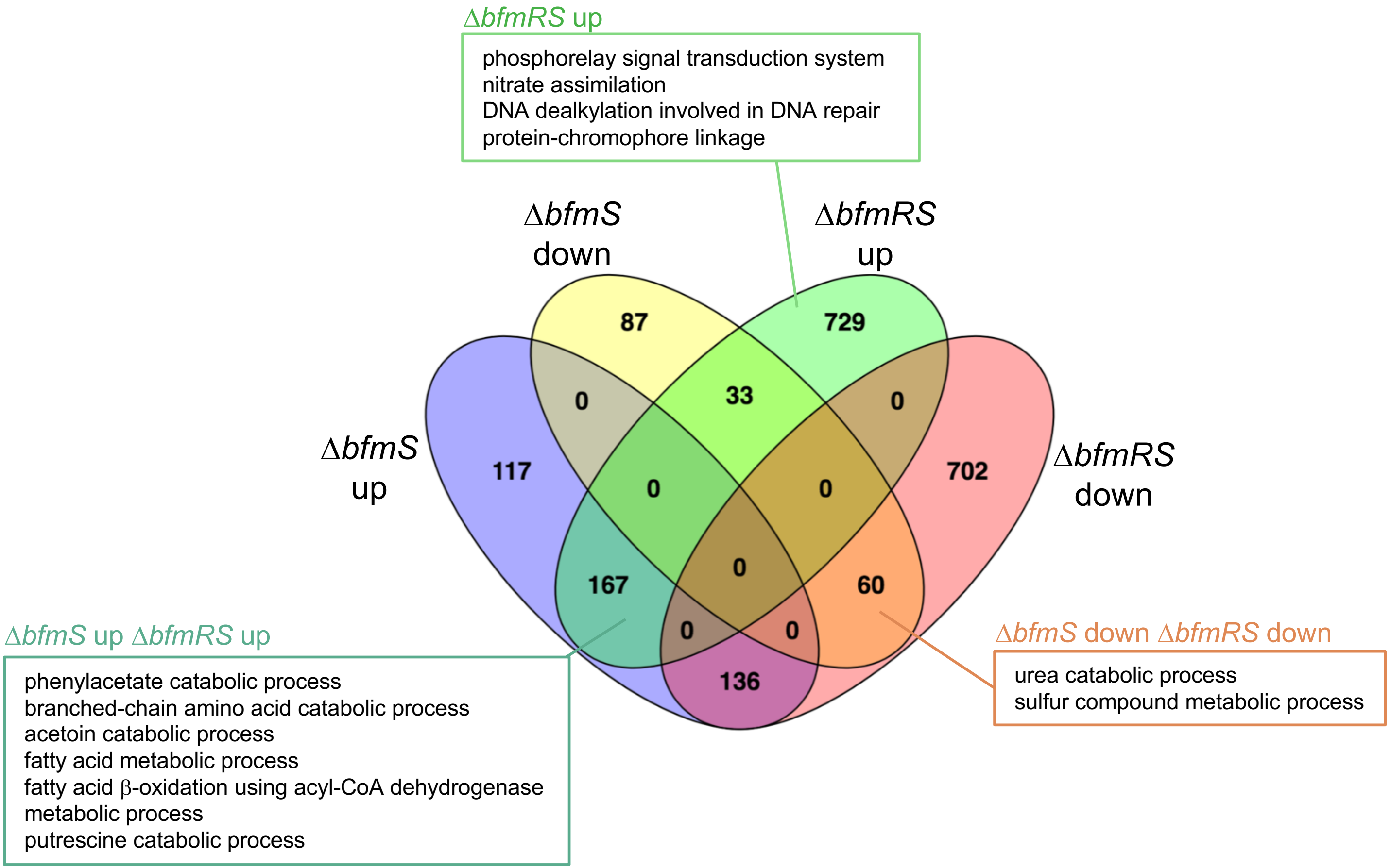
ΔbfmRS up
phosphorelay signal transduction system
nitrate assimilation
DNA dealkylation involved in DNA repair
protein-chromophore linkage
ΔbfmS down
ΔbfmRS up
ΔbfmS up
ΔbfmRS down
87
729
0
33
0
117
0
0
702
0
167
60
0
0
136
ΔbfmS up ΔbfmRS up
phenylacetate catabolic process
branched-chain amino acid catabolic process
acetoin catabolic process
fatty acid metabolic process
fatty acid β-oxidation using acyl-CoA dehydrogenase
metabolic process
putrescine catabolic process
ΔbfmS down ΔbfmRS down
urea catabolic process
sulfur compound metabolic process
