# Supplementary material for: A global regulatory system links virulence and antibiotic resistance to envelope homeostasis in Acinetobacter baumannii
Source: PLoS Pathog. 2018 May 24;14(5):e1007030. doi: 10.1371/journal.ppat.1007030 (PMC5967708; doi:10.1371/journal.ppat.1007030)
Supplement: S1 Table — (PDF) [file ppat.1007030.s009.pdf]

S1 Table. Strains and plasmids.

Strain or plasmid	Genotype or description	Reference
A. baumannii		
ATCC 17978	cerebrospinal fluid isolate	[1]
EGA195	ATCC 17978 Δ*bfmS*::*aacC1* (Gm^R)	[2]
EGA496	ATCC 17978 Δ*bfmR*::*aacC1*	this study
EGA495	ATCC 17978 Δ*bfmRS*::*aacC1*	this study
EGA497	ATCC 17978 Δ*bfmRS*::*aacC1*::pEGE152	this study
EGA501	ATCC 17978 Δ*bfmRS*::*aacC1*::pEGE224	this study
EGA507	ATCC 17978 Δ*bfmRS*::*aacC1*::pEGE234	this study
EGA465	ATCC 17978 Δ*adeIJK*	this study
EGA513	ATCC 17978 Δ*adc*	this study
EGA514	ATCC 17978 Δ*oxa51*	this study
EGA515	ATCC 17978 Δ*adc* Δ*oxa51*	this study
EGA516	ATCC 17978 Δ*ampG*	this study
EGA517	ATCC 17978 Δ*ampG* Δ*bfmRS::aacC1*	this study
EGA518	ATCC 17978 Δ*ampG* Δ*bfmS::aacC1*	this study
EGA624	ATCC 17978 Δ*adc* Δ*bfmS::aacC1*	this study
EGA534	ATCC 17978 Δ*bfmRS*::*aacC1* *cspC*(G17A*fs*X21)	this study
EGA552	ATCC 17978 Δ*bfmRS*::*aacC1* *ACX60_RS05385* (NUDIX)::*ISAba1*	this study
EGA555	ATCC 17978 Δ*bfmRS*::*aacC1* *mnmA*(G362S)	this study
EGA692	ATCC 17978 Δ*pbp2*	this study
EGA694	ATCC 17978 Δ*pbp2* Δ*bfmS*::*aacC1*	this study
EGA695	ATCC 17978 Δ*pbp2* Δ*bfmRS*::*aacC1*	this study
EGA68	ATCC 17978 ΔKL3::*aacC1*	[2]
EGA127	ATCC 17978 $bfmS^{1\text{-}467}$ (G467DfsX19)	[2]
EGA187	ATCC 17978 $bfmS^{1\text{-}467}$ (G467DfsX19) ΔKL3::*aacC1*	this study
ATCC 19606	urine isolate	[1]
EGA216	ATCC 19606 Δ*bfmS*::*aacC1*	[2]
AB5075-UW	bone isolate/osteomyelitis	[3]
AB08299	AB5075-UW *bfmS*::T26	[3]
E. coli		
DH5α	*supE44* Δ*lacU169* (ϕ80lacZΔM15) *hsdR17 recA1 endA1 gyrA96 thi*-1 *relA1*	[4]
DH5λpir	DH5α (λpir) *tet*::Mu *recA*	[5]
TO60	DH5α λpir [F′ *proAB* $lacI^q$ZΔM15 Tn10 (Tc^R)]	[6]
**plasmids**		
pUC18	*oriColE1* MCS Cb^R	[7]
pSR47s	*oriTRP4 oriR6K* Km^R	[8]
pEGE148	pSR47s::*aacC1* Km^R Gm^R	[2]
pEGE152	pEGE148::*bfmR bfmS*	[2]
pEGE224	pEGE148::*bfmR bfmS*(H324Q, 3XFLAG)	this study
pEGE234	pEGE148::*bfmR*(3XFLAG)	this study
pJB1801	oriRSF1010 Cb^R	[2]
pEGE144	pJB1801::*bfmR bfmS*	this study

pEGE146	pJB1801::*bfmR bfmS*(G494V)	this study
pWH1266	shuttle plasmid, Tc^R Cb^R	[9]
pFPV25	promoter trap vector containing promoterless *gfp*mut3	[10]
pEGE244	pWH1266 with *rrnB*,T7 tandem terminators replacing EcoRI-PstI fragment, Tc^R	this study
pEGE245	pEGE244 with SacI-PstI fragment of pFPV25	this study
pEGE313	pEGE245 with *adc*p inserted upstream of *gfp*mut3	this study
pEGE314	pEGE245 with *oxa51*p inserted upstream of *gfp*mut3	this study
pEGE315	pEGE245 with *slt*p inserted upstream of *gfp*mut3	this study
pEGE253	pEGE245 with *ygeR*p inserted upstream of *gfp*mut3	this study
pEGE254	pEGE245 with *tolB*p inserted upstream of *gfp*mut3	this study
pEGE246	pEGE245 with *ACX60_RS18040*p inserted upstream of *gfp*mut3	this study
pEGE247	pEGE245 with *ompW*(*ACX60_RS05905*)p inserted upstream of *gfp*mut3	this study
pEGE248	pEGE245 with *ACX60_RS13710*p inserted upstream of *gfp*mut3	this study
pEGE251	pEGE245 with *ACX60_RS09685*p inserted upstream of *gfp*mut3	this study
pEGE304	pWH1266 with EcoRI-PstI fragment replaced by polylinker, Tc^R	this study
pCA24N-*dinB*	Source of *lacI*^{q},T5-*lac*p fragment	[11, 12]
pEGE305	pEGE304 with *lacI*^{q},T5-*lac*p fragment, Tc^R	this study
pEGE316	pEGE305 with *slt* gene downstream of T5-*lac*p promoter	this study

References

1. Bouvet P, Grimont P. Taxonomy of the Genus Acinetobacter with the Recognition of Acinetobacter baumannii sp. nov. Acinetobacter haemolyticus sp. nov. Acinetobacter johnsonii sp. nov. and Acinetobacter junii sp. nov. and Emended Descriptions of Acinetobacter calcoaceticus and Acinetobacter lwofii. International Journal of Systematic Bacteriology. 1986;36(2):228-40.
2. Geisinger E, Isberg RR. Antibiotic modulation of capsular exopolysaccharide and virulence in Acinetobacter baumannii. PLoS Pathog. 2015;11(2):e1004691. Epub 2015/02/14. doi: 10.1371/journal.ppat.1004691 PPATHOGENS-D-14-02426 [pii]. PMID: 25679516.
3. Gallagher LA, Ramage E, Weiss EJ, Radey M, Hayden HS, Held KG, et al. Resources for Genetic and Genomic Analysis of Emerging Pathogen Acinetobacter baumannii. J Bacteriol. 2015;197(12):2027-35. doi: 10.1128/JB.00131-15. PMID: 25845845.
4. Hanahan D, Jessee J, Bloom FR. Plasmid transformation of Escherichia coli and other bacteria. Methods Enzymol. 1991;204:63-113. Epub 1991/01/01. doi: 0076-6879(91)04006-A [pii]. PMID: 1943786.
5. Kolter R, Inuzuka M, Helinski DR. Trans-complementation-dependent replication of a low molecular weight origin fragment from plasmid R6K. Cell. 1978;15(4):1199-208. Epub 1978/12/01. doi: 0092-8674(78)90046-6 [pii]. PMID: 728998.
6. O'Connor TJ, Adepoju Y, Boyd D, Isberg RR. Minimization of the Legionella pneumophila genome reveals chromosomal regions involved in host range expansion. Proc Natl Acad Sci U S A. 2011;108(36):14733-40. PMID: 21873199.
7. Yanisch-Perron C, Vieira J, Messing J. Improved M13 phage cloning vectors and host strains: nucleotide sequences of the M13mp18 and pUC19 vectors. Gene. 1985;33(1):103-19. Epub 1985/01/01. PMID: 2985470.
8. Andrews HL, Vogel JP, Isberg RR. Identification of linked Legionella pneumophila genes essential for intracellular growth and evasion of the endocytic pathway. Infect Immun. 1998;66(3):950-8. PMID: 9488381.
9. Hunger M, Schmucker R, Kishan V, Hillen W. Analysis and nucleotide sequence of an origin of DNA replication in Acinetobacter calcoaceticus and its use for Escherichia coli shuttle plasmids. Gene. 1990;87(1):45-51. PMID: 2185139.
10. Valdivia RH, Falkow S. Bacterial genetics by flow cytometry: rapid isolation of Salmonella typhimurium acid-inducible promoters by differential fluorescence induction. Mol Microbiol. 1996;22(2):367-78. PMID: 8930920.
11. Kitagawa M, Ara T, Arifuzzaman M, Ioka-Nakamichi T, Inamoto E, Toyonaga H, et al. Complete set of ORF clones of Escherichia coli ASKA library (a complete set of E. coli K-12 ORF archive): unique resources for biological research. DNA Res. 2005;12(5):291-9. Epub 2006/06/14. doi: 10.1093/dnares/dsi012. PMID: 16769691.
12. Macguire AE, Ching MC, Diamond BH, Kazakov A, Novichkov P, Godoy VG. Activation of phenotypic subpopulations in response to ciprofloxacin treatment in Acinetobacter baumannii. Mol Microbiol. 2014;92(1):138-52. Epub 2014/03/13. doi: 10.1111/mmi.12541. PMID: 24612352.
