# Supplementary material for: A global regulatory system links virulence and antibiotic resistance to envelope homeostasis in Acinetobacter baumannii
Source: PLoS Pathog. 2018 May 24;14(5):e1007030. doi: 10.1371/journal.ppat.1007030 (PMC5967708; doi:10.1371/journal.ppat.1007030)
Supplement: S2 Table — (PDF) [file ppat.1007030.s010.pdf]

S2 Table. Oligonucleotide primers

primer name	Sequence (5' – 3'; restriction site underlined, with partial sites in italics)	RE site(s)
cloning		
H234Q-F	*AG*AACTCAGAACACCTGTGGCACG	SmlI
H234Q-R	*TGAG*ATACTGCTCTCATTAACTCACGTTG	SmlI
bfmS-Rkpn	CTGAGGTACCGCAAACCTATTTTGGAACCTGATG	KpnI
3xFLAGtop	*G*GGATCCGTCGACTCTAGAGGCGGTGACTACAAAGACCATGACGGTGATTAT AAAGATCATGATATTGACTACAAAGATGACGACGATAAATAGTAA*GGTAC*	PstI, BamHI, XbaI, KpnI
3xFLAGbottom	*C*TTACTATTTATCGTCGTCATCTTTGTAGTCAATATCATGATCTTTATAATCACCGT CATGGTCTTTGTAGTCACCGCCTCTAGAGTCGACGGATCC*CTGCA*	PstI, BamHI, XbaI, KpnI
bfmR-3FLAG-F	*TGA*TATTGACTACAAAGATGACGACGATAAATAAAATCTGATTAAACTTCCTATAA GGTTGG	BspHI
bfmR-3FLAG-R	*TGA*TCTTTATAATCACCGTCATGGTCTTTGTAGTCACCGCCCAATCCATTGGTTTC TTTAACAAACAAGTAAC	BspHI
DadeIJK-upR3	AAAGGAGCTCCGACATCATTGTTCCACCTCG	Sac
DadeIJK-upF3	TAGAGGATCCTCACATTGCCTTTGCGTCAGC	BamHI
DadeIJK-dwnF	GGTAGAGCTCCCATCTAGTGCTGAACTTAAAAAGCAATAAG	SacI
DadeIJK-dwnR2	AGCAGTCGACTTATTCGGATTGAACAAGACCAAGC	SalI
Dadc-upR	CTAAAGGTACCAAGCGGGGATAAAAGTAGACAAG	KpnI
Dadc-upF	GCTGGGATCCACGGAAAAAGAAGGTGATTGG	BamHI
Dadc-dwnF	GCATTGGTACCGCTTATGCTGTGCTGAATGC	KpnI
Dadc-dwnR3	CAAGGGTCGACCAAAGCTCCTGTGTATCGAC	SalI
Doxa51-upR	CGCTGGTACCAAGTAAGAGTGCTTTAATGTTCATAAGAC	KpnI
Doxa51-upF	GGTGGGATCCGTGAAAGAGCCAATAGAAGC	BamHI
Doxa51-dwnF	ATTAGGTACCTTATAGAGTTAGTTTATAGCCCAGTTCAC	KpnI
Doxa51-dwnR	GAGAGTCGACTCCTACGGCAGTTGTTCTTG	SalI
pbp2-upF	ATCAGGATCCATTTGGCTCACCACCATTTCAC	BamHI
pbp2-upR	TTTAAGGTACCGTGCTGTTTCATACGGATCGAC	KpnI
pbp2-dwnF	CTCCAGGTACCACACCAACTCGTCCTGCTACAAAC	KpnI
pbp2-dwnR	ATTCAGTCGACTGAGAAGCAAGGGCTGTTTAAGG	SalI
DampG-up-R	AGACGGTACCAGAGGTTTGTGTGGTCATTCG	KpnI
DampG-up-F	TTTAAGGATCCTTCAAGGCCTTTCACTTGC	BamHI
DampG-dwn-F	AGATGGTACCACACATGACATAGAAAAGGCAGG	KpnI
DampG-dwn-R	AAGGGTCGACAACTCACCTGATGCCAAAGC	SalI
rrnBT7TT	GATCATGAATTCCAGGCATCAAATAAAACGAAAGGCTCAGTCGAAAGACTGGGC CTTTCGTTTTATCTGTTGTTTGTCGGTGAACGCTCTCTACTAGAGTCACACTGGCT CACCTTCGGGTGGGCCTTTCTGCGTTTATAGAGCTCATACCTGCAGAGTCAT	EcoRI, SacI, PstI
slt-F	CCTAAAGAATTCTAGTCAGGATTTAACGCATTTATGAGACC	EcoRI
slt-R	TTATGCCTGCAGAAAGAATGTTCCATCATTCATCATCTAAAAC	PstI
adc-F	CATCATGAGCTCCCATTCTATGGCCGTGTTC	SacI
adc-R	TAAATCTCTAGATTAGCTCATAAATTATTTAAAAAGAAAGATGCCTAC	XbaI
oxa-F	TAAAACGAGCTCTATCTCAAAAAGAAAATTCATTGCAG	SacI
oxa-R	TTAATGTCTAGAAGACTTATAAAATTGAATTTGAAGTTTAGC	XbaI
sltp-F	TAAAATGAGCTCAAACGGGTCAAACCAATCATTATAGCAG	SacI
sltp-R	TCTTGTTCTAGAAGCTCTTTTTACCTGGTCTCATAAATGC	XbaI
ygeRp-F	CTTACCGAGCTCGCCGTGCCCAATCTAAAC	SacI
ygeRp-R	CAGTTATCTAGATCGATCTCCCTCACTAAATGCTAC	XbaI
tolBp-F	CTAAAAGAGCTCAAGAAGAAGCTGCCCAGA	SacI
tolBp-R	CTCGTTTCTAGAGGTTTAAAGTCATTGAAGGTATTTTTGT	XbaI
RS09685p-F	CTGTGGGAGCTCTGGGGTTGTGGTCTTAGGTG	SacI

RS09685p-R	CATTTTTCTAGATTCTCCCTAGCCCAAATGTTACTC	XbaI
lipo1804pSF	AGCTTAGAGCTCGGCGCTAAAGTGG	SacI
lipo1804pXR	AATTTTTTCTAGATAGCTTTCCTTTCATTACGACAAAC	XbaI
ompW05pSF	GGCAAAGAGCTCTGCTTGGTGACAAATGGACAG	SacI
ompW05p-XR	CTTTTTCTCTAGAAAAGGACTCCATGTCCGAGC	XbaI
gp1371pSF	CAATCAGGAGCTCAAGCGGCTCAAATCAACAAG	SacI
gp1371pXR	TAGCTATCTAGATTTTATGGCATCCTTAAATTCATTA	XbaI

qRT-PCR		
ftsZ-qF1	CCAGGTAGCAGCAGAAGAAA	
ftsZ-qR1	TACCTGTACCACCACCCATA	
LT_17-qF1	AACTTGTGCTGGTAACTTGTATG	
LT_17-qR1	CTGTCGCTTTGAACCAACTATC	
ygeR-qF2	GGTCCGTTCAGGTGATACTTTA	
ygeR-qR2	GCGGTGCAATACCATTCATC	
pbp3-qF2	AGATAAGCGAACAAAGCAAACA	
pbp3-qR2	CCGCCCAGAGCAGATAAA	
slt70-qF1	CACTAGGCCGTTTAGCAAATAAT	
slt70-qR1	GGCTACGGTTCGATAGAGATAC	
tolB-qF1	ATGGGCAAATGGTGGTTTATG	
tolB-qR1	ACCTTGCTCACTCGGTAAAT	
tolQ-qF3	GATCAGGGTAGTCTGGAATATGG	
tolQ-qR3	AACAGCAGCCAAACCAATAAA	
adc-qF1	GTATGGCTGTGGGTGTTATTC	
adc-qR1	TACTGACAGAACCTAGCTCAAA	
oxa51-qF1	GTGAAGCGTGTTGGTTATGG	
oxa51-qR1	GCCTCTTGCTGAGGAGTAAT	
uppP-qF1	AGTGATCCAGTTAGGTGCTATT	
uppP-qR1	TGACTCGACATCACCACTAAA	
uppS-qF1	GCTATGGTGGTATGTGGGATA	
uppS-qR1	AGCTGGCAGATCGTTTAGA	
ybjG-qF1	GGTGTGCATTTCCCTTTAGAC	
ybjG-qR1	ACCGTAAGCATTCCAGAAGTA	
lolA-qF1	CAGTGCTTGCTCCTGTAATG	
lolA-qR1	GTATTCGCTTTCGTTGTCTGT	
16S_qF	CAGCTCGTGTCGTGAGATGT	
16S_qR	CGTAAGGGCCATGATGACTT	
rpoC-qF4	CAAACGGTGAGCCAATCATC	
rpoC-qR4	GCCTTCACCTTTCGCATTT	
