# Supplementary material for: A global regulatory system links virulence and antibiotic resistance to envelope homeostasis in Acinetobacter baumannii
Source: PLoS Pathog. 2018 May 24;14(5):e1007030. doi: 10.1371/journal.ppat.1007030 (PMC5967708; doi:10.1371/journal.ppat.1007030)
Supplement: S3 Table — (PDF) [file ppat.1007030.s011.pdf]

S3 Table. Minimal Inhibitory Concentrations (µg/ml) determined from colony formation efficiency assays testing *bfmRS* mutants.

	**WT**	**Δ*bfmS***	**Δ*bfmR***	**Δ*bfmRS***	**Δ*bfmRS*/*bfmRS*⁺**
vancomycin	500	500		500	500
rifampicin	4	4		1	4
erythromycin	16	16	4	4	16
colistin	1	1	0.5	0.5	1
ciprofloxacin	0.5	0.5	0.125	0.125	0.5
tobramycin	2	2	0.5	0.5	2
amikacin	4	8	2	2	4
chloramphenicol	128	64	128	128	128
phosphomycin	125	125		250	
A22	64	64		64	64
mecillinam	64	256		2	64
imipenem	0.4	0.4	0.025	0.025	0.4
ampicillin	64	128		8	
carbenicillin	16	32		4	16
cefoxitin	64	64		8	
cephalexin	400	>400		50	400
aztreonam	16	64		4	
ceftazidime	4	16		1	
sulbactam	0.5	2		0.5	
