# Supplementary material for: A global regulatory system links virulence and antibiotic resistance to envelope homeostasis in Acinetobacter baumannii
Source: PLoS Pathog. 2018 May 24;14(5):e1007030. doi: 10.1371/journal.ppat.1007030 (PMC5967708; doi:10.1371/journal.ppat.1007030)
Supplement: S4 Table — (PDF) [file ppat.1007030.s012.pdf]

S4 Table. Minimal Inhibitory Concentrations (µg/ml) determined from colony formation efficiency assays testing β-lactamase mutants.

	WT	Δ*adc*	Δ*oxa51*	Δ*adc*Δ*oxa51*
mecillinam	64	16	64	16
imipenem	0.4	0.4	0.4	0.4
ampicillin	64	16	64	16
carbenicillin	8	8	8	8
cephalexin	400	100	400	100
aztreonam	16	16	16	8
ceftazidime	4	4	4	4
sulbactam	0.5	0.5	0.5	0.5
