# Supplementary material for: A global regulatory system links virulence and antibiotic resistance to envelope homeostasis in Acinetobacter baumannii
Source: PLoS Pathog. 2018 May 24;14(5):e1007030. doi: 10.1371/journal.ppat.1007030 (PMC5967708; doi:10.1371/journal.ppat.1007030)
Supplement: S5 Table — (PDF) [file ppat.1007030.s013.pdf]

S5 Table. Derivatives of Δ*bfmRS* containing mutations allowing suppression of antibiotic hypersensitivity.

Strain name	antibiotic used for isolation (µg/ml)[a]	mutation	position	annotation[b]	locus ID	protein ID	gene	description
**Regulators**								
EGA534	IPM 0.025	Δ1 bp	1,349,273	coding (45/213 nt)	*ACX60_RS06250* →	WP_000126912.1	*cspC*	cold-shock protein
EGA590	IPM 0.05	C→T	1,349,412	Q62* (CAA→TAA)	*ACX60_RS06250* →	WP_000126912.1	*cspC*	cold-shock protein
EGA587	IPM 0.05	ISAba11 insertion		intergenic (-256/-119)	*ACX60_RS06245* / *ACX60_RS06250*	WP_000126912.1	*cspC*	oxidoreductase / cold-shock protein
EGA610	IPM 0.05	Δ1 bp	2,069,424	coding (184/273 nt)	*ACX60_RS09765* ←	WP_001043034.1	*hupB*	DNA-binding protein HU-beta
EGA610 (2nd mutation)	IPM 0.05	Δ95 bp	1,412,371	coding (411-505/1203 nt)	*ACX60_RS06590* ←	WP_005135012.1		MFS transporter
EGA636	MEC 8	Δ9 bp	813,574	coding (22-30/288 nt)	*ACX60_RS03925* ←	WP_000887099.1		YbeD/DUF493 domain-containing protein
**Nucleotide hydrolysis**								
EGA552	MEC 8	ISAba11 insertion		coding (278/489 nt)	*ACX60_RS05385*	WP_000741966.1		NUDIX hydrolase
**tRNA synthesis**								
EGA652	MEC 8	C→A	1,509,411	P57T (CCG→ACG)	*ACX60_RS07030* →	WP_000803938.1	*glnS*	glutamine--tRNA ligase
EGA659	MEC 8	G→A	1,509,516	D92N (GAT→AAT)	*ACX60_RS07030* →	WP_000803938.1	*glnS*	glutamine--tRNA ligase
EGA540	MEC 8	C→T	1,509,526	P95L (CCG→CTG)	*ACX60_RS07030* →	WP_000803938.1	*glnS*	glutamine--tRNA ligase
EGA549	MEC 8	C→A	1,509,526	P95Q (CCG→CAG)	*ACX60_RS07030* →	WP_000803938.1	*glnS*	glutamine--tRNA ligase
EGA546	MEC 8	C→T	1,509,714	R158C (CGT→TGT)	*ACX60_RS07030* →	WP_000803938.1	*glnS*	glutamine--tRNA ligase
EGA668	MEC 8	A→C	1,510,018	Q259P (CAA→CCA)	*ACX60_RS07030* →	WP_000803938.1	*glnS*	glutamine--tRNA ligase
EGA543	MEC 8	C→T	1,510,093	S284F (TCT→TTT)	*ACX60_RS07030* →	WP_000803938.1	*glnS*	glutamine--tRNA ligase
EGA600	IPM 0.05	C→T	2,816,359	G423D (GGC→GAC)	*ACX60_RS13385* ←	WP_002017216.1	*lysS*	lysine--tRNA ligase
EGA641	MEC 8	A→G	2,816,837	F264L (TTC→CTC)	*ACX60_RS13385* ←	WP_002017216.1	*lysS*	lysine--tRNA ligase
EGA642	MEC 8	+C	3,051,007	coding (1604/2064 nt)	*ACX60_RS14470*	WP_001226779.1	*metG*	methionine--tRNA ligase
EGA575	IPM 0.175	G→A	3,544,293	noncoding (61/76 nt)	*ACX60_RS16715* ←			tRNA-Thr
EGA562	IPM 0.175	G→A	3,544,298	noncoding (56/76 nt)	*ACX60_RS16715* ←			tRNA-Thr
EGA574	IPM 0.175	G→A	3,544,305	noncoding (49/76 nt)	*ACX60_RS16715* ←			tRNA-Thr
EGA583	IPM 0.175	C→T	3,544,308	noncoding (46/76 nt)	*ACX60_RS16715* ←			tRNA-Thr
EGA579	IPM 0.175	T→C	3,544,331	noncoding (23/76 nt)	*ACX60_RS16715* ←			tRNA-Thr
EGA576	IPM 0.175	T→C	3,544,333	noncoding (21/76 nt)	*ACX60_RS16715* ←			tRNA-Thr
EGA578	IPM 0.175	G→A	3,544,343	noncoding (11/76 nt)	*ACX60_RS16715* ←			tRNA-Thr
**tRNA modification**								
EGA555	MEC 8	C→T	1,148,445	G362S (GGC→AGC)	ACX60_RS05380	WP_001187604.1	*mnmA*	tRNA 2-thiouridine(34) synthase
EGA646	MEC 8	+A	1,148,726	coding (803/1134 nt)	ACX60_RS05380	WP_001187604.1	*mnmA*	tRNA 2-thiouridine(34) synthase
EGA556	MEC 8	Δ15 bp	1,148,779	coding (736-750/1134 nt)	ACX60_RS05380	WP_001187604.1	*mnmA*	tRNA 2-thiouridine(34) synthase
EGA637	MEC 8	G→A	1,148,796	R245C (CGT→TGT)	ACX60_RS05380	WP_001187604.1	*mnmA*	tRNA 2-thiouridine(34) synthase

EGA655	MEC 8	T→G	1,148,942	D196A (GAT→GCT)	ACX60_RS05380	WP_001187604.1	*mnmA*	tRNA 2-thiouridine(34) synthase
EGA588	IPM 0.05	$(T)_{6\rightarrow 7}$	1,148,949	coding (580/1134 nt)	ACX60_RS05380	WP_001187604.1	*mnmA*	tRNA 2-thiouridine(34) synthase
EGA666	MEC 8	A→G	1,148,969	L187P (CTA→CCA)	ACX60_RS05380	WP_001187604.1	*mnmA*	tRNA 2-thiouridine(34) synthase
EGA591	IPM 0.05	T→G	1,149,248	N94T (AAC→ACC)	ACX60_RS05380	WP_001187604.1	*mnmA*	tRNA 2-thiouridine(34) synthase
EGA663	MEC 8	T→C	1,149,426	K35E (AAA→GAA)	ACX60_RS05380	WP_001187604.1	*mnmA*	tRNA 2-thiouridine(34) synthase
EGA650	MEC 8	ISAba11 insertion		coding (588 nt)	ACX60_RS05380	WP_001187604.1	*mnmA*	tRNA 2-thiouridine(34) synthase
EGA532	IPM 0.025	$(TTATCG)_{2\rightarrow 3}$	1,426,039	coding (37/1881 nt)	ACX60_RS06660	WP_000559185.1	*gidA / mnmG*	tRNA uridine-5-carboxymethylaminomethyl(34) synthesis enzyme
EGA635	MEC 8	Δ1 bp	1,667,927	coding (144/312 nt)	*ACX60_RS07825* ←	WP_001047539.1	*tusE*	sulfite reductase
EGA630	MEC 8	T→A	1,667,961	H37L (CAT→CTT)	*ACX60_RS07825* ←	WP_001047539.1	*tusE*	sulfite reductase
EGA592	IPM 0.05	C→T	1,668,020	W17* (TGG→TGA)	*ACX60_RS07825* ←	WP_001047539.1	*tusE*	sulfite reductase
EGA664	MEC 8	ISAba11 insertion		coding (11 nt)	*ACX60_RS07825* ←	WP_001047539.1	*tusE*	sulfite reductase
EGA629	MEC 8	ISAba11 insertion		coding (307nt)	*ACX60_RS07825* ←	WP_001047539.1	*tusE*	sulfite reductase
EGA651	MEC 8	Δ1 bp	1,668,935	coding (221/369 nt)	*ACX60_RS07840* ←	WP_000105719.1	*tusD*	sulfurtransferase
**Ribosome synthesis/activity**								
EGA535	IPM 0.175	G→A	450,408	A58T (GCT→ACT)	*ACX60_RS02180* →	WP_001141025.1	*rpsE*	30S ribosomal protein S5
EGA536	IPM 0.175	C→A	453,415	H23Q (CAC→CAA)	*ACX60_RS02210* →	WP_001040166.1	*rpsK*	30S ribosomal protein S11
EGA565	IPM 0.175	A→G	445,018	intergenic (+10/-4)	*ACX60_RS02110* → / → *ACX60_RS02115*	WP_001122317.1/ WP_001138119.1	*rplB / rpsS*	50S ribosomal protein L2/30S ribosomal protein S19
EGA573	IPM 0.175	C→T	446,989	Q61* (CAG→TAG)	*ACX60_RS02135* →	WP_000849928.1	*rpmC*	50S ribosomal protein L29
EGA537	IPM 0.175	Δ189 bp	2,002,396	coding (368-556/1005 nt)	*ACX60_RS09440* ←	WP_000441015.1	*prmB*	ribosomal protein L3 N(5)-glutamine methyltransferase
EGA561	IPM 0.175	G→A	2,002,570	Q128* (CAA→TAA)	*ACX60_RS09440* ←	WP_000441015.1	*prmB*	ribosomal protein L3 N(5)-glutamine methyltransferase
EGA571	IPM 0.175	ISAba11 insertion		coding (896/1005 nt)	*ACX60_RS09440*	WP_000441015.1	*prmB*	ribosomal protein L3 N(5)-glutamine methyltransferase
EGA631	MEC 8	C→A	1,282,031	intergenic (+46/-92)	*ACX60_RS05915* → / → *ACX60_RS05920*	WP_001982118.1/ WP_000125378.1		30S ribosomal protein S2/elongation factor Ts
EGA551	MEC 8	(TGC)4→3	1,282,419	coding (297-299/876 nt)	*ACX60_RS05920* →	WP_000125378.1	*tsf*	elongation factor Ts
EGA648	MEC 8	A→T	2,961,703	V386E (GTA→GAA)	*ACX60_RS14035*	WP_001029610.1	*tuf*	elongation factor Tu
EGA632	MEC 8	C→G	2,962,283	A193P (GCT→CCT)	*ACX60_RS14035*	WP_001029610.1	*tuf*	elongation factor Tu
EGA634	MEC 8	G→C	2,962,740	Y40* (TAC→TAG)	*ACX60_RS14035*	WP_001029610.1	*tuf*	elongation factor Tu
EGA541	MEC 8	Δ2 bp	2,962,760	coding (99-100/1191 nt)	*ACX60_RS14035*	WP_001029610.1	*tuf*	elongation factor Tu
EGA644	MEC 8	G→C	2,962,771	A30G (GCT→GGT)	*ACX60_RS14035*	WP_001029610.1	*tuf*	elongation factor Tu
EGA550	MEC 8	T→C	2,962,787	K25E (AAA→GAA)	*ACX60_RS14035*	WP_001029610.1	*tuf*	elongation factor Tu
EGA547	MEC 8	T→C	2,962,795	D22G (GAC→GGC)	*ACX60_RS14035*	WP_001029610.1	*tuf*	elongation factor Tu
EGA645	MEC 8	C→T	2,962,814	G16S (GGT→AGT)	*ACX60_RS14035*	WP_001029610.1	*tuf*	elongation factor Tu
EGA643	MEC 8	A→G	2,962,858	M1T (ATG→ACG) †	*ACX60_RS14035*	WP_001029610.1	*tuf*	elongation factor Tu
EGA539	IPM 0.175	Δ27 bp	3,481,676	coding (607-633/2700 nt)	*ACX60_RS16410* ←	WP_000130326.1	*infB*	translation initiation factor IF-2

**Peptidoglycan synthesis**								
EGA593	IPM 0.05	C→T	317,172	G728S (GGT→AGT)	*ACX60_RS01520* ←	WP_000736677.1	*pbp1A*	penicillin-binding protein 1A
**isoprenoid synthesis**								
EGA564	IPM 0.175	C→T	801,341	E93K (GAA→AAA)	*ACX60_RS03850* ←	WP_000234078	*ispB*	solanesyl diphosphate synthase
EGA577	IPM 0.175	+TAA	801,646	intergenic (-29/+19)	*ACX60_RS03850* ← / ← *ACX60_RS03855*	WP_000234078	*ispB*	solanesyl diphosphate synthase/hypothetical protein
**Peptidase**								
EGA595	IPM 0.05	ISAba11 insertion		coding (22/1185 nt)	*ACX60_RS16915*	WP_000939111.1	*ctpA*	peptidase S41
EGA598	IPM 0.05	Δ9 bp	3,143,075	coding (527-535/1452 nt)	*ACX60_RS14880* ←	WP_005135781.1		peptidase C13 family protein
EGA603	IPM 0.05	Δ2 bp	3,142,683	coding (926-927/1452 nt)	*ACX60_RS14880* ←	WP_005135781.1		peptidase C13 family protein
**Unknown**								
EGA567	IPM 0.175	ISAba11 insertion		coding (251/300 nt)	*ACX60_RS03855*	WP_000667115.1		hypothetical protein
EGA570	IPM 0.175	ISAba11 insertion		coding (126/300 nt)	*ACX60_RS03855*	WP_000667115.1		hypothetical protein
EGA580	IPM 0.175	Δ1 bp	3,008,574	coding (138/561 nt)	*ACX60_RS14290* ←	WP_001275712.1		hypothetical protein
EGA633	MEC 8	ISAba11 insertion		coding (348 nt)	ACX60_RS06190	WP_000409701.1		
EGA647	MEC 8	Δ30 bp	1,668,450	coding (264-293/351 nt)	*ACX60_RS07835* ←	WP_000859774.1		hypothetical protein
EGA656	MEC 8	ISAba1 insertion		coding (343 nt)	ACX60_RS07835	WP_000859774.1		hypothetical protein
EGA658	MEC 8	ISAba1 insertion		coding (348 nt)	ACX60_RS07835	WP_000859774.1		
EGA667	MEC 8	G→A	1,668,368	Q3* (CAA→TAA)	*ACX60_RS07830* ←	WP_000194629.1		hypothetical protein

a. Δ*bfmRS* bacteria were spread on LB agar containing imipenem (IPM) or mecillinam (MEC) at the indicated concentrations, and resistant mutants were purified on LB agar.

b. Description of the mutation given in *breseq* output. From *breseq* 0.30.0 documentation: "For base substitutions inside genes, shows the resulting amino acid and codon changes. For other mutations inside genes, gives the local coordinates of the nucleotides affected. For mutations in intergenic regions, gives two relative positions (e.g., +150/-119) where the numbers are the distances from the mutation to the nearest neighboring genes before and after it in the genome, and the +/- signs indicate whether the mutation is oriented upstream or downstream with respect to each of these genes." http://barricklab.org/twiki/pub/Lab/ToolsBacterialGenomeResequencing/documentation/index.html
